# Chlamydial Infection From Outside to Inside

**DOI:** 10.3389/fmicb.2019.02329

**Published:** 2019-10-09

**Authors:** Arlieke Gitsels, Niek Sanders, Daisy Vanrompay

**Affiliations:** ^1^Laboratory for Immunology and Animal Biotechnology, Department of Animal Sciences and Aquatic Ecology, Faculty of Bioscience Engineering, Ghent University, Ghent, Belgium; ^2^Laboratory of Gene Therapy, Department of Nutrition, Genetics and Ethology, Faculty of Veterinary Medicine, Ghent University, Merelbeke, Belgium

**Keywords:** *Chlamydia*, internalization, pathogen–host cell interactions, inclusion membrane proteins, vesicular pathways, non-vesicular pathways

## Abstract

*Chlamydia* are obligate intracellular bacteria, characterized by a unique biphasic developmental cycle. Specific interactions with the host cell are crucial for the bacteria’s survival and amplification because of the reduced chlamydial genome. At the start of infection, pathogen-host interactions are set in place in order for *Chlamydia* to enter the host cell and reach the nutrient-rich peri-Golgi region. Once intracellular localization is established, interactions with organelles and pathways of the host cell enable the necessary hijacking of host-derived nutrients. Detailed information on the aforementioned processes will increase our understanding on the intracellular pathogenesis of chlamydiae and hence might lead to new strategies to battle chlamydial infection. This review summarizes how chlamydiae generate their intracellular niche in the host cell, acquire host-derived nutrients in order to enable their growth and finally exit the host cell in order to infect new cells. Moreover, the evolution in the development of molecular genetic tools, necessary for studying the chlamydial infection biology in more depth, is discussed in great detail.

## Introduction

**FIGURE 1:**
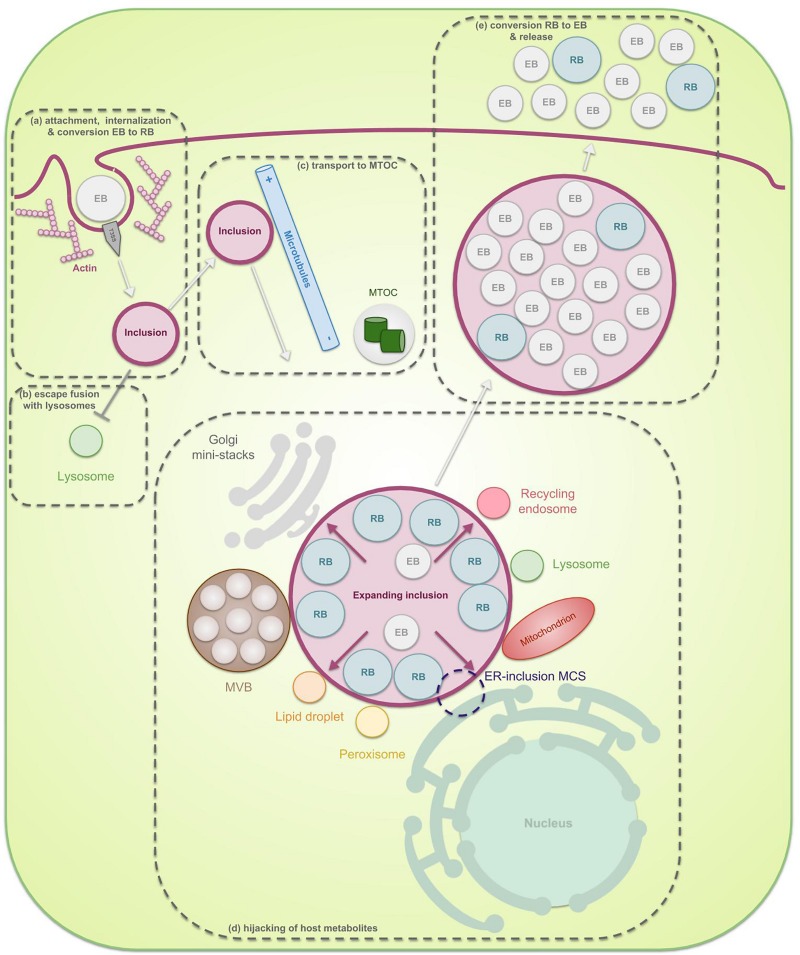
The chlamydial developmental cycle in light of membrane interaction. Chlamydiae are characterized by their unique biphasic life cycle in which they alternate between two morphological forms: EBs and RBs. **(a)** At the start of infection, extracellular infectious EBs use bacterial ligands to bind receptors on the surface of the host cell. The attachment subsequently enables internalization of the bacteria into a vesicle inside the host cell, called the inclusion. Internalization can be both dependent or independent of actin but since the actin-dependent process has been studies more extensively, this is the one depicted in the figure. Herein, EBs inject pre-packed T3SS effectors into the host cytoplasm as soon as contact with the host is established, leading to reorganization of actin and uptake of the EBs. Next, the EBs, residing inside the inclusion, transition into RBs. RBs are metabolically active particles which are able to amplify through binary fission. **(b)** These RBs immediately produce early effectors which modify the inclusion membrane in order to prevent lysosomal degradation. **(c)** Furthermore, the inclusion starts traveling across microtubules away from the periphery and towards the microtubule organizing center (MTOC). **(d)** Once the inclusion reaches the nutrient-rich peri-Golgi region, the pathogen hijacks host cell metabolites in order to support its own growth as well as the growth of the inclusion membrane, necessary to allow room for the expanding RBs. Nutrients are obtained through specific interactions of the inclusion with multiple host cell organelles such as fragmented Golgi mini-stacks, the ER, lipid droplets, peroxisomes, lysosomes, recycling endosomes, mitochondria, and multivesicular bodies (MVBs). **(e)** Finally, the expanded inclusion fills up most of the host cell cytoplasm after which the RBs transition back to EBs. These then exit the host cell in order to infect new cells.

Chlamydiae are known for their unique biphasic life cycle during which they alternate between two morphological forms: infectious extracellular elementary bodies (EBs) and metabolically active intracellular reticulate bodies (RBs). It is clear that multiple membranes have a key function in the chlamydial developmental cycle (Figure 1). In order to enter the host cell, naked EBs must surpass its plasma membrane. Therefore, they attach to the host cell membrane by means of bacterial ligands and host receptors, and inject pre-packaged effectors inside the host cytosol which enable invasion. During internalization of the EBs, a vacuole, called the inclusion, is formed in which the EBs reside and promptly transform into RBs (Hackstadt, 2012; Hegemann and Mölleken, 2012; Mehlitz and Rudel, 2013). Multiple EBs can bind and enter the same host cell, leading to multiple inclusions inside this cell. For some species, like *Chlamydia (C.) trachomatis*, these inclusions ultimately fuse by means of homotypic fusion, creating one large inclusion (Elwell and Engel, 2012; Hackstadt, 2012; Richards et al., 2013). The latter process is orchestrated by the inclusion membrane protein (Inc) IncA and will be discussed in more detail later in this review (see “SNARE proteins”). Metabolically active RBs quickly modify the inclusion membrane by means of early effectors in order to prevent degradation of the inclusion and to enable transport of the inclusion toward the microtubule-organizing center (MTOC) (Scidmore et al., 1996). When located at the nutrient-rich peri Golgi region, chlamydial mid-cycle gene products further modify the inclusion membrane, enabling selective interactions with cellular compartments and pathways in order to hijack essential nutrients (Moore and Ouellette, 2014). Since the chlamydial genome is substantially reduced (ca. 1 Mb, encoding 895 open reading frames for *C. trachomatis*) and lacks many metabolic enzymes, survival of the pathogen depends on these interactions (Stephens et al., 1998). However, the inclusion membrane forms a barrier between the pathogen and the host’s nutrients, meaning that mechanisms for nutrient transport across the inclusion membrane are also membrane-related processes which are crucial to the survival of chlamydiae. Furthermore, RBs multiply inside of the inclusion by means of binary fission and the inclusion expands. The lipids that are needed for the expansion of the inclusion membrane and the amplification of the RBs are scavenged from the host (Hackstadt et al., 1995, 1996; Scidmore et al., 1996; Wylie et al., 1997; Van Ooij et al., 2000; Carabeo et al., 2003; Su et al., 2004). The proteins present in those membranes are mostly *Chlamydia*-specific (Taraska et al., 1996). Finally, at the end of their life cycle, RBs transition back into EBs in an asynchronous manner after which these EBs exit the host cell in order to infect new cells.

It should be mentioned that the survival and growth of *Chlamydia* not only depends on the establishment of an intracellular niche, from where it can hijack a myriad of host cell nutrients, but also on the evasion of the immune response. When successful, the latter phenomenon not only saves the bacterium from clearance but also effectuates an asymptomatic course of chlamydial infections. The employed strategies differ between species since different chlamydial strains infect various hosts with each their specific types of immune responses. Several groups devoted their research on studying the molecular mechanisms driving these immune evasion strategies. For example, *C. trachomatis* was shown to paralyze the host immune system by preventing the activation of polymorphic nuclear leukocytes (Rajeeve et al., 2018), was proven to avoid a human-specific, ubiquitin-mediated marking of the inclusion for destruction (Haldar et al., 2016), to affect host antigen presentation by increasing the presentation of self-antigen and thereby decreasing the presentation of *Chlamydia*-derived peptides (Cram et al., 2016; Pickering et al., 2017). Besides its immune evasion strategies, *Chlamydia* has evolved an escape to certain stressors by switching to a physiological state in which the bacterium ceases to divide but remains viable, called persistence. We would like to refer to Panzetta et al. (2018) and Chen et al. (2019) who reviewed the molecular mechanisms *Chlamydia* employs to counteract host innate immune defenses as well as to establish persistence (Panzetta et al., 2018; Chen et al., 2019).

At present, the events occurring at the host plasma membrane as well as at the inclusion membrane supporting the internalization and nourishing of chlamydiae will be discussed in more detail. It should be noted that since there are significant species- and strain-specific differences in the way that *Chlamydia* interacts with the host cell, data cannot always be extrapolated to other *Chlamydia* species and caution should be exercised (Valdivia, 2008). In addition, at the end of this review, recent advances in the development of molecular genetic tools, necessary for studying these chlamydial processes in more depth, are discussed. By bundling all the recent data on the chlamydial life cycle in light of membrane interactions as well as on recently discovered advances in promising molecular genetic manipulation techniques for *Chlamydia*, the authors aim to assist scientists in identifying novel strong targets for *Chlamydia* prophylactics and therapeutics.

## Chlamydial Transport Through the Host Cell

### Attachment

**FIGURE 2:**
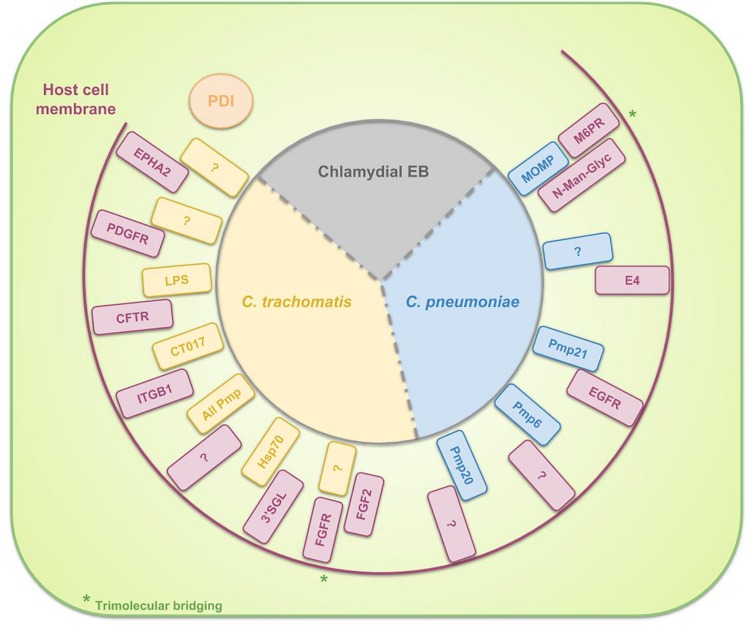
Schematic representation of the binding partners, involved in the binding of EBs to the surface of host cells. For *C. trachomatis* three complete binding partners are identified: chlamydial LPS and host Cystic Fibrosis Transmembrane Conductance Regulator (CFTR), chlamydial CT017 and host beta-1 integrin (ITGB1) and chlamydial heat shock protein 70 (Hsp70) and host 3′sulfogalactolipid (3′SGL). Furthermore, several chlamydial adhesins or host receptors are known to be involved in the attachment of *C. trachomatis* EBs to the host cell without knowing their binding partners: host Ephrin receptor A2 (EPHA2), Platelet-derived growth factor receptor (PDGFR) and Fibroblast growth factor receptor (FGFR) (by means of Fibroblast growth factor 2 (FGF2) bridging) and all chlamydial polymorphic membrane proteins (Pmps). For *C. pneumoniae* the identified binding couples to date are chlamydial Pmp21 and host Epidermal growth factor receptor (EGFR) and chlamydial MOMP and host mannose 6-phosphate receptor (M6PR) (by means of *N*-Man-Glyc bridging). Host PDI is necessary for EB attachment to the cell but the bacterium does not bind directly to the PDI. Instead, *Chlamydia* attaches to a host protein, associated with PDI.

Differences between species in host- and tissue-tropism are in part due to the diversity in binding and internalization mechanisms. The attachment of *Chlamydia* to the host cell is assumed to be a multistep process. The initial interaction of *C. trachomatis* and *C. pneumoniae* EBs with the host cell appears to be a reversible, low-affinity electrostatic interaction (Heckels et al., 1976; Hatch et al., 1981) of the EB with host heparan sulfate-like glycosaminoglycans (GAGs) (Zhang and Stephens, 1992; Su et al., 1996). Examples of known EB ligands to bind heparan sulfate GAGs are OmcB for *C. pneumonia* (Mölleken and Hegemann, 2008) and *C. trachomatis* (Fadel and Eley, 2008) and major outer membrane protein (MOMP) for *C. trachomatis* (Su et al., 1996). Furthermore, by chemically mutating Chinese hamster ovary (CHO) cells and next selecting the clones that are resistant to chlamydial infection, the group of Carabeo and Hackstadt discovered a previously undescribed irreversible, temperature-dependent and heparin-resistant binding step, occurring subsequent to the reversible binding of *C. trachomatis* serovar L2 EBs to cell-surface heparan sulfate. This binding step was proven to be crucial for L2 infection and moreover, to differentiate the lymphogranuloma venereum (LGV) biovar (of which the L2 serovar is a member) from the trachoma biovars (Carabeo and Hackstadt, 2001). Moreover, Fudyk et al. (2002) complemented these results and proved that specific mutations modify the infectivity of *C. trachomatis* LGV differently compared to trachoma biovars or *C. pneumoniae*. Furthermore, based on their results of infecting mutated CHO cell lines with *C. trachomatis* serovars as well as well as *C. pneumoniae*, they hypothesized that chlamydiae utilize a common multistep internalization pathway with specific requirements per species (Fudyk et al., 2002). In the meantime, several bacterial adhesins and host receptors are identified which are involved in the attachment of EBs to the host cell. These are listed in Table 1 and depicted in Figure 2. Some binding partners remain unidentified and probably more molecular interactions will exist but aren’t discovered yet. Moreover, host Protein Disulfide Isomerase (PDI), a component of the estrogen receptor complex, is shown to have a dual function in the entry of chlamydiae into the cell. On the one hand, cell-surface PDI-mediated disulfide reduction allows entry and on the other hand structural PDI allows attachment, independent of PDI enzymatic activity. In the latter case, *Chlamydia* binds to a host protein that is associated with PDI instead of binding directly to cell-associated PDI (Abromaitis and Stephens, 2009). Furthermore, chlamydiae use the involvement of PDI in attachment and invasion by controlling its activity in order to avoid re-infection. When an infected cell gets re-infected during the replicative phase of the RBs, the formation of EBs may be prevented. *C. trachomatis* therefore induces the depletion of Glucose regulated protein 96 (Gp96) from the infected cells during its replicative phase, resulting in reduced activity of PDI on the cell surface (Karunakaran et al., 2015).

**TABLE 1:** Overview of the binding partners, involved in the binding of EBs to the surface of host cell for specific chlamydial species.

**Chlamydial adhesin**	**Host receptor**	**Species**	**References**
LPS	Cystic fibrosis transmembrane conductance regulator (CFTR)	*C. trachomatis*	Ajonuma et al., 2010
MOMP (CT681)	mannose 6-phosphate receptor/insulin-like growth factor receptor 2 (M6PR/IGFR2) via N-Man-Glyc	*C. pneumoniae*	Puolakkainen et al., 2005
CT017 (Ctad1)	Beta-1 integrin (ITGB1)	*C. trachomatis*	Stallmann and Hegemann, 2016
Pmp21	Epidermal growth factor receptor (EGFR/ERBB)	*C. pneumoniae*	Mölleken et al., 2013
Hsp70	3′sulfogalactolipid (3′SGL)	*C. trachomatis*	Mamelak et al., 2001
?	Fibroblast growth factor receptor (FGFR) via Fibroblast growth factor 2 (FGF2)	*C. trachomatis*	Kim et al., 2011
?	Platelet-derived growth factor receptor (PDGFR)	*C. trachomatis*	Elwell et al., 2008
?	Ephrin receptor A2 (EPHA2)	*C. trachomatis*	Subbarayal et al., 2015
?	Apolipoprotein E4	*C. pneumoniae*	Gerard et al., 2008
All Pmps	?	*C. trachomatis*	Becker and Hegemann, 2014
Pmp6	?	*C. pneumoniae*	Mölleken et al., 2010
Pmp20	?	*C. pneumoniae*	Mölleken et al., 2010

As shown in Table 1 and Figure 2, all polymorphic membrane proteins (Pmps) are involved in the attachment of *C. trachomatis* to the host cell. The Pmp family is the largest protein family of *Chlamydia* species and it is a unique feature of the genus (Horn et al., 2004; Vandahl et al., 2004). Since the Pmps account for 3.15 and 5.1% of the total coding capacity of *C. trachomatis* and *C. pneumoniae*, respectively (Grimwood and Stephens, 1999), which is a relatively high proportion of the greatly reduced genome, it is suggested that the Pmps might play an important role in chlamydial biology. *In silico* predictions identify Pmps as autotransporter (type V secretion system) proteins, based on their cleavable N-terminal signal sequence for translocation across the inner membrane, their central passenger domain which provides the protein’s function, their β-barrel-shaped C-terminal transporter domain and the phenylalanine at the end of this domain, suggesting outer membrane localization (Struyve et al., 1991; Henderson and Lam, 2001; Dautin and Bernstein, 2007). Moreover, experimental evidence of this *in silico* prediction has been generated for several Pmps (Longbottom et al., 1998; Vandahl et al., 2002; Wehrl et al., 2004; Kiselev et al., 2007; Liu et al., 2010). One of the functions of these autotransported proteins is that of adhesin, as the conserved motifs GGA (I,L,V) and FxxN were also found in adhesins of *Anaplasma phagocytophilum* (Girard and Mourez, 2006). Mölleken et al. (2010) showed that yeast cells, expressing *C. pneumoniae* Pmp6, Pmp20 and Pmp21 (orthologs of PmpG, PmpB and PmpD of *C. trachomatis*, respectively) on their surface, and beads coated with recombinant proteins of these three Pmps adhere to human epithelial cells. The observation that pre-incubation of epithelial cells with these three proteins reduced the binding significantly confirmed the adhesive capacities of Pmp6, Pmp20, and Pmp21 of *C. pneumoniae*. Pmps appear to be functioning as species-specific adhesins, as incubation of human epithelial and endothelial cells with *C. trachomatis* Pmps was not effective to block a subsequent *C. pneumoniae* infection and *vice versa*, suggesting that Pmps are involved in host and tissue tropism (Becker and Hegemann, 2014). The size and amino acid sequences of the Pmps are highly variable. The number of Pmp genes depends on the species (Vasilevsky et al., 2016), ranging from 9 to 16 full length genes for the chlamydial reference strains *C. abortus* S26/3 (Thomson et al., 2005), *C. avium* 10DC88 (Sachse et al., 2014), *C. caviae* GPIC (Read, 2003), *C. felis* FE/C-56 (Azuma et al., 2006), *C. gallinacea* 08-1274/3 (Sachse et al., 2014), *C. muridarum* Nigg (Read et al., 2000), *C. pecorum* DBDeUG (Bachmann et al., 2014), *C. pneumoniae* CWL029 (Kalman et al., 1999), *C. psittaci* ATCC VR-125/6BC (Voigt et al., 2011), and *C. trachomatis* D/UW-3/Cx (Stephens et al., 1998). Given the fact that the family of Pmps is most probably a product of gene duplications, enabling functional diversity, it is assumed that these proteins are directly linked to the variations in disease severity, observed between different strains (Abdelsamed et al., 2013). For example, although Van Lent et al. (2016) observed structural similarities between *C. psittaci* Pmps and therefore suggested comparable functions, they identified *C*. *psittaci* PmpA and PmpH as important players in pathogenesis. Moreover, the apparent immunoaccessibility/antigenicity of these two Pmps suggested their potential in vaccine design (Van Lent et al., 2016). Gomes et al. (2006) studied polymorphisms in the nine Pmps of all *C. trachomatis* serovars and delivered evidence for the correlation of the identified genetic variations with tissue tropism. More specifically, analysis of these polymorphisms showed the strongest relation between the LGV serovars, causing invasive urogenital diseases, who differed the most from the ocular and non-LGV urogenital serovars. Furthermore, phylogenetic reconstructions demonstrated that for six of the nine Pmp genes, the serovars clustered based on tissue tropism. Finally, they provided statistically significant evidence for intergenomic recombination between Pmp genes, possibly enabling evolutionary adaptations in tissue tropism and pathogenesis (Gomes et al., 2006). Moreover, the group also proved the differential occurrence of putative insertion sequences among Pmps of different serovars, representing distinct disease or prevalence groups (Gomes et al., 2004). Lastly, Jeffrey et al. (2010) proved the correlation between polymorphisms in the Pmp genes across *C. trachomatis* serovars and rectal tropism. Thus, in conclusion, polymorphisms in Pmps relate to strain distinction, tissue tropism and possibly disease severity.

### Internalization

A myriad of research groups has devoted their resources in studying the internalization of chlamydiae but despite all of the data gathered over the years, still no consensus pathway has been described and a lot of conflicting results were published over the years. For example, there is still a controversy about the contribution of clathrin-mediated endocytosis to the internalization of chlamydiae (Hodinka et al., 1988; Wyrick et al., 1989; Majeed and Kihlström, 1991). Microscopy studies have both supported and invalidated the concept of receptor-mediated endocytosis by clathrin-coated pits: although three research groups observed association of *C. trachomatis* with clathrin-coated pits and/or the uptake of *C. trachomatis* strains into clathrin-coated vesicles (Hodinka et al., 1988; Wyrick et al., 1989; Majeed and Kihlström, 1991), other researchers showed that *C. trachomatis* entry was unaltered when clathrin-dependent endocytosis was inhibited (Ward and Murray, 1984; Boleti et al., 1999). The latter could be supported by the fact that conventional clathrin pits measure 100 nm in diameter whilst chlamydial EBs have an average size of 250 nm (Hybiske and Stephens, 2007). Similarly, research on caveola-mediated entry has provided both supporting (Norkin et al., 2001; Stuart et al., 2003; Webley et al., 2004) and disproving (Gabel et al., 2004) evidence. Moreover, studies provide evidence of *Chlamydia* entering the cell through directed phagocytosis (Byrne and Moulder, 1978) as well as through generalized pinocytosis (Reynolds and Pearce, 1990).

**FIGURE 3:**
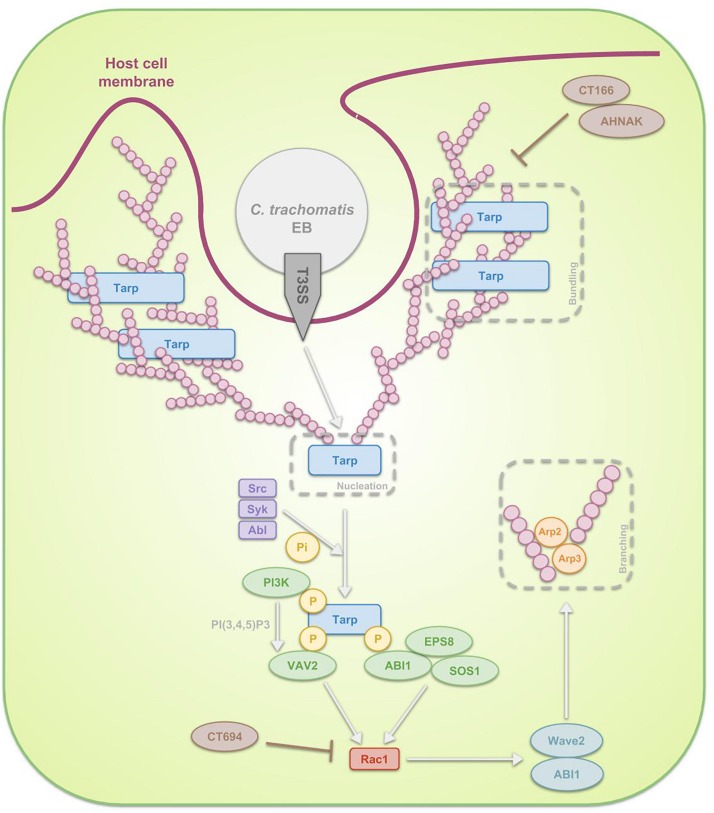
Schematic representation of the signal transduction pathways, involved in the internalization of *C. trachomatis* EBs through phagocytosis. After attachment of the EB to the host cell, activation of the T3SS takes place. The first T3SS effectors that get secreted are Tarp, CT166 and CT694. Tarp reorganizes actin in a direct as well as in an indirect manner. The first mechanism reflects the fact that Tarp contains actin-binding domains and a proline-rich region, enabling Tarp respectively to be a nucleator and to enhance actin bundling. The indirect process on the other hand is a Rac1-dependent mechanism. Host Src, Syk and Abl kinases phosphorylate the N-terminal tyrosine-rich tandem repeats of Tarp, leading to the recruitment of several proteins. On the one hand ABI1 interacts with the phosphorylated Tarp and complexes with SOS1 and EPS8. On the other hand, PI3K binds phosphorylated Tarp, producing PI(3,4,5)P3 and thus activating another protein which also interacts with the phosphorylated Tarp, VAV2. Subsequently SOS1 and VAV2 activate Rac1, which next recruits WAVE2, ABI1, ARP2, and ARP3. These actin regulators finally reorganize actin. Since Tarp-induced actin rearrangements are transient, it is believed that CT166 and CT694 regulate the actin de-polymerization.

However, overall, investigations focused predominantly on actin driven processes (Figure 3) whereat EBs carry a functional type 3 secretion system (T3SS) through which, upon attachment, they translocate pre-packaged effectors along with their respective chaperones directly into the host cell cytoplasm in order to induce the cytoskeletal rearrangements (Peters et al., 2007; Saka et al., 2011). Prior to attachment of chlamydiae to the host, the activity of this completely assembled T3SS in EBs is prevented by means of disulfide bonding within Secretion and cellular translocation protein (Sct) F and SctC, both structural proteins of the T3SS, and by means of positioning a CopN plug on the cytoplasmic face of the T3SS. The mentioned CopN plug consists of a complex of CopN with T3SS chaperones Scc1 and Scc4 N-terminally, positioning CopN at the T3SS of EBs (Silva-Herzog et al., 2011) and with additional chaperone Scc3 C-terminally, which inhibits the secretion of CopN (Slepenkin et al., 2005; Ferrell and Fields, 2016). Immediately after the EBs attach to the host cell plasma membrane, the T3SS gets activated though the secretion of CopN and deployment of translocator proteins CopB and CopD. CopB and CopD then form the invasion-related translocon that enables the transport of subsequently secreted T3S effectors (Bulir et al., 2014, 2015; Ferrell and Fields, 2016). Gene duplication were detected for CopB and CopD, called CopB2 and CopD2. The group of Chellas-Géry et al. (2011) compared CopB to CopB2 and showed that, although both localize to the inclusion membrane, CopB2 was continuously detectable whilst CopB was only detectable at some points during the infection, being early on and 20 h post infection. Therefore, one could argue the possibility of CopB mediating early and late translocation whereas CopB2 functioning in the meantime (Chellas-Géry et al., 2011).

The activated T3SS secretes invasion-related effectors, effectuating the internalization process. In case of *C. trachomatis*, translocated actin-recruiting phosphoprotein (Tarp), CT166 and CT694 are secreted first (Pais et al., 2013; Chen et al., 2014). Tarp is an early multidomain effector protein that mediates actin nucleation and bundling. Two mechanisms are described by which Tarp exerts its effects. Firstly, Tarp is in direct contact with actin: on the one hand it is a nucleator, considering it contains several C-terminal actin-binding domains with similarity to WH2 domain proteins and on the other hand it contains a proline-rich region that may enhance actin oligomerization (Jewett et al., 2006). Secondly, Tarp enables actin remodeling through a RAS-related C3 botulinum toxin substrate 1 (Rac1)-dependent mechanism at the attachment sites (Carabeo et al., 2004; Clifton et al., 2004). In the latter scenario, host Src (Jewett et al., 2008), Syk (Mehlitz et al., 2008), and Abl (Elwell et al., 2008) kinases phosphorylate the N-terminal tyrosine-rich tandem repeats of Tarp, leading to the recruitment of son of sevenless homologue 1 (SOS1) and VAV2. Interaction between SOS1 and phosphorylated Tarp is mediated through ABL interactor 1 (ABI1), which forms a multiprotein complex with SOS1 along with epidermal growth factor receptor kinase substrate 8 (EPS8). Activation of VAV2 on the other hand is dependent on phosphatidylinositol-3,4,5-triphosphate (PI(3,4,5)P3), produced by phosphoinositide 3-kinase (PI3K), an enzyme also binding phosphorylated Tarp. SOS1 and VAV2 activate Rac1, which in turn recruits the actin regulators Wiskott-Aldrich syndrome protein family member 2 (WAVE2, also known as WASF2), ABI1, actin-related protein 1 (ARP2) and ARP3. These regulators are essential for actin reorganization (Carabeo et al., 2007; Lane et al., 2008; Jiwani et al., 2012; Mehlitz and Rudel, 2013). The actin polymerization is accompanied by extensive membrane remodeling. Actin rearrangements induced by Tarp are transient and it is hypothesized that other effectors, such as *C. trachomatis* CT166 (Thalmann et al., 2010) and CT694 (Hower et al., 2009) might regulate the actin de-polymerization. Whereas *C. trachomatis* recruits only Rac1 and not Cdc42, *C. caviae* has been show to use both in addition to Arf6 in order to promote its entry (Subtil et al., 2004; Balañá et al., 2005). As far as we know, the role of Arf6 during *C. trachomatis* infection has not been examined yet.

Clifton et al. (2005) analyzed the genomes of *C. trachomatis* serovar L2 and D, *C. muridarum*, *C. caviae* and *C. pneumoniae* and discovered that each contained an ortholog of Tarp. Moreover, no phosphotyrosine was detected at the site of entry for the orthologs from *C. muridarum*, *C. caviae* and *C. pneumoniae*. However, all of these orthologs contain at least one and up to four functional C-terminal actin binding domains and for each of the species, purified recombinant Tarps were capable of nucleating actin filament formation *in vitro* (Jewett et al., 2010). This indicates the dependence of actin recruitment on the presence of the C-terminal domain of Tarp and not necessarily on tyrosine phosphorylation (Clifton et al., 2005). Moreover, phylogenetic analysis of Tarp from *C. trachomatis* reference strains as well as ocular, genital and LGV *C. trachomatis* clinical isolates resulted in a clustering of LGV and ocular isolates, separated from a cluster formed by the urogenital isolates. Also, LGV and ocular strains could easily be distinguished based on the number of tyrosine-rich repeats (up to nine for LGV strains whilst only one for ocular strains) and the number of actin binding domains (two for LGV strains whilst up to four for ocular strains) for Tarp. This suggests that, besides the previously mentioned *C. trachomatis* Pmps, also Tarp could plausibly play a role in *C. trachomatis* adaptations to a specific niche within the host (Lutter et al., 2010). Nevertheless, although the genes encoding Pmps and Tarp, among others, have been shown to group *C. trachomatis* serovars based on tissue tropism, classification of *C. trachomatis* strains in serovars is not based on Tarp or Pmps but on MOMP. MOMP is an immunodominant surface antigen which function is on the one hand a porin in RBs (Bavoil et al., 1984) and on the other hand a potential adhesin in EBs (Su et al., 1990; Su and Caldwell, 1991). However, the phylogenetic categorization of MOMP is not concordant with pathobiotypes or tissue tropism of *C. trachomatis* (Stothard et al., 1998; Millman et al., 2006). Therefore, analyzing other genetic variations between the different *C. trachomatis* strains, aside of those present in the gene encoding MOMP, might allow the identification of new factors that enable specific tissue tropisms or disease severity. However, since comparison of the complete genome of an oculotropic isolate with a genitotropic isolate pointed out a 99.6% identity (Carlson et al., 2005), it is clear that host genetic differences will also influence chlamydial disease outcome (Abdelsamed et al., 2013).

### Intracellular Transportation to the MTOC

**FIGURE 4:**
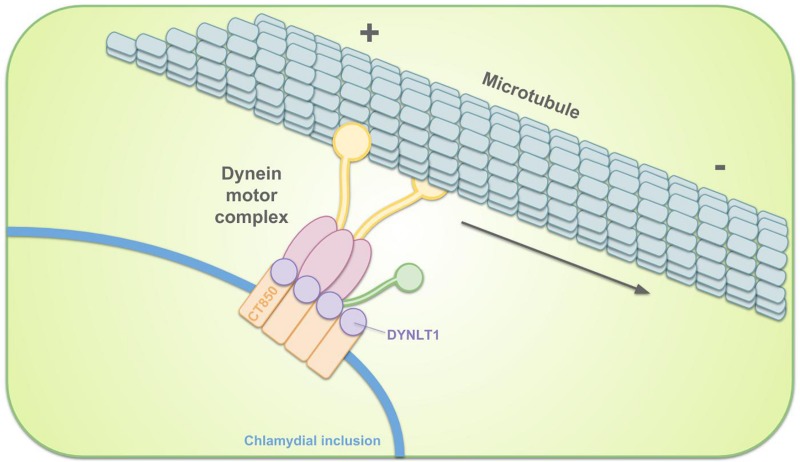
Schematic representation of the intracellular transportation of the chlamydial inclusion toward the MTOC. Remodeling of the inclusion membrane enables dynein-dependent migration of the inclusion along microtubules. *C. trachomatis* inclusion membrane protein CT850 can directly bind to DYNLT1 to promote the positioning of the inclusion at the MTOC.

The EBs are, immediately after entry, sequestered in the inclusion. Remodeling of the inclusion membrane by insertion of bacterial proteins quickly dissociates it from the endosomal pathway, avoiding lysosomal fusion (Scidmore et al., 1996; Scidmore et al., 2003). The remodeled membrane subsequently promotes migration of the inclusion along microtubules to the MTOC, nearby the peri-Golgi region (Scidmore et al., 1996; Grieshaber et al., 2003). Localization to the MTOC facilitates interactions with nutrient-rich compartments (Richards et al., 2013). This transport is dynein-dependent and p50 dynamitin-independent. However, it is incorrect to state that transportation is dynactin-independent since some components of the dynactin complex still get recruited (Hackstadt, 2012; Kokes and Valdivia, 2012). For example, Sherry et al. (2018) showed that dynactin interacts with *C. trachomatis* Inc CT192 during infection and is recruited to the inclusion membrane in a CT192-dependent manner. p50 dynamitin usually links cargo to microtubules. It is therefore suggested that chlamydial effector proteins in the inclusion membrane mimic the cargo-binding activity in order to tether the inclusion to dynein and/or centrosomes (Grieshaber et al., 2003). *C. trachomatis* infection was shown to increase activation of Src family kinases (SFKs) and these SFKs were proven to be required for microtubule-dependent trafficking of the inclusion to the MTOC and for intracellular growth. Migration to the MTOC is absent in *C. caviae* and *C. muridarum*, who do not recruit SFKs. Moreover, an increase in inclusion development and bacterial growth when inhibiting SFKs suggested that SFKs restrict growth of these non-human strains (Mital et al., 2010; Mital and Hackstadt, 2011a). *C. trachomatis* Incs (discussed in more detail in the section on vesicular pathways) IncB (also known as CT232), CT101, CT222, and CT850 reside in cholesterol-rich microdomains at the point of centrosome–inclusion contact and colocalize with active SFKs (Mital et al., 2010), making it likely that these Incs participate in transport. For example, *C. trachomatis* CT850 directly binds to dynein light chain 1 (DYNLT1) to enable the trafficking of the inclusion to the MTOC (Mital et al., 2015) (Figure 4). In case of *C. psittaci*, IncB has been shown to interact with Snapin, which also binds dynein, thus connecting the inclusion to the microtubule network (Böcker et al., 2014). Snapin is a cytoplasmic host protein with a multivalent role in intracellular trafficking (Lu et al., 2009).

### Stabilizing the Inclusion Membrane

The inclusion membrane is extremely fragile, hence its only recently successful purification (Aeberhard et al., 2015). In order to preserve its integrity and structural stability, F-actin and intermediate filaments encapsulate the inclusion in the form of a dynamic scaffold (Kumar and Valdivia, 2008; Bastidas et al., 2013). Since disruption of inclusion integrity leads to leakage of inclusion contents into the host cytoplasm and a robust activation of IL-8 expression, the cytoskeletal scaffold on the inclusion could limit exposure of bacterial products to cytoplasmic innate immune surveillance pathways (Buchholz and Stephens, 2008; Kumar and Valdivia, 2008). Recruitment and assembly of F-actin takes place by means of RhoA GTPases (Kumar and Valdivia, 2008), septins (Volceanov et al., 2014), EGFR signaling (Patel et al., 2014), and CT813 (Kokes et al., 2015). Kumar and Valdivia proposed a model wherein *C. trachomatis* Incs recruit RhoA, which in turn triggers F-actin assembly. F-actin next recruits and stabilizes intermediate filament proteins through linker molecules, resulting in the formation of a stable scaffold around the inclusion. The necessary flexibility of this structure surrounding an expanding inclusion would then be provided by CPAF, a chlamydial protease that progressively cleaves the preassembled filaments without loss of their structural function (Kumar and Valdivia, 2008). Additionally, microtubules reorganize at the inclusion surface in order to create a microtubule superstructure. However, since this superstructure is responsible for the reorganization of Golgi mini-stacks, the recruitment and assembly of this cytoskeletal scaffold will be discussed in more detail in the section on chlamydial interaction with the Golgi apparatus.

Furthermore, *C. trachomatis* inclusions show membrane deformation and tubulation. Mirrashidi et al. (2015) showed that this morphology was effectuated by IncE which recruited sorting nexin (SNX) proteins SNX5 and SNX6. More specifically, the C-terminal region of IncE interacted with the phox-homology (PX) domains of SNX5 or SNX6 (Mirrashidi et al., 2015). These SNX proteins provide a mechanistic link to tubulation, as they are necessary and sufficient for membrane deformation and tubule formation (Frost et al., 2009; van Weering et al., 2012). Finally, Aeberhard et al. (2015) proved that the SNX protein recruitment by *C. trachomatis* is not a means to enable bacterial infections but rather to circumvent SNX protein-enhanced bacterial destruction, since SNX proteins regulate endocytic and lysosomal degradation. This statement was based on the fact that RNAi-mediated depletion of SNX5/SNX6 did not slow down infection but rather increased the production of infectious *C. trachomatis* progeny (Aeberhard et al., 2015).

### Release

Knowledge on the mechanism of chlamydial release from infected cells is limited. Banks et al. (1970) proved in the year 1970 the induced lysis and concomitant host cell death by egressing *C. trachomatis* of the LGV biovar and later, Todd and Caldwell (1985) showed that the ocular and genital biovars of *C. trachomatis* exit cells without associated death of the host cell.

In the year 2007 two research groups published a paper on the release of *C. trachomatis* EBs from the host cell (Beatty, 2007; Hybiske and Stephens, 2007). Remarkably, both papers showed strongly contradictory conclusions. Beatty complemented the aforementioned paper of Todd and Caldwell (1985) by clarifying the mechanism of release. She showed that the egression of *C. trachomatis* serovar E (thus a genital biovar) was mediated by the expansion of the intracellular bacterial inclusion, accompanied by the disruption of the plasma membrane integrity. Exocytic fusion of the lysosomes with the plasma membrane was promoted by the calcium-induced actin depolymerization. She then also explained that associated host cell death was avoided by a lysosomal-mediated reparative process in which lysosomes are delivered to the plasma membrane. Importantly, this repair also supported chlamydial persistency because of the retention of left-over bacteria within the surviving host cell (Beatty, 2007). The second paper on chlamydial egress from the host cell, published in 2007, was a paper form Hybiske and Stephens. In this paper, two mutually exclusive release methods were discussed: lysis and extrusion. Lytic egress was described as a destructive mode of release, proceeding by a temporally well-defined two-step process: first the cysteine protease-dependent lysis of the inclusion vacuole, followed by the rupturing of the host cell plasma membrane. The latter process appeared to be regulated by intracellular calcium signaling. Furthermore, Nguyen et al. (2018) identified an interaction between the Inc MrcA and the Ca^2+^ channel inositol-1,4,5-trisphosphate receptor type 3 (ITPR3) and proved through mutagenesis and siRNA depletion studies that Ca^2+^ signaling pathways are involved in the regulation of *C. trachomatis* release. In contrast, extrusion represented the actin-dependent package release of portions of chlamydial inclusions by means of membranous protrusions. Both the original cell and the residual inclusion would remain intact thus promoting persistence of the infection. Hence, release of inflammatory content would be prevented and EBs would be protected from host cell immunity. The released EBs would finally reside in extracellular inclusion bodies, surrounded by the actin cytoskeleton (Chin et al., 2012), the host plasma membrane, and a thin layer of cytoplasm between the plasma and inclusion membranes. Extrusion was proven to be dependent on actin polymerization, neuronal Wiskott-Aldrich syndrome protein, myosin II and RhoA. The latter specifically regulated the final stage of extrusion, being the pinching off of the inclusion body (Hybiske and Stephens, 2007). Furthermore, results showed that survival of *C. trachomatis* was significantly lower when infecting macrophages or DCs compared to epithelial cells (Steele et al., 2004). However, the group of Hybiske has proven that bone marrow macrophages as well as DCs engulf extrusions and that subsequent survival of chlamydiae is hence promoted by the lipid barrier surrounding them (Sherrid and Hybiske, 2017; Zuck et al., 2017). Nevertheless, the engulfment of extrusions initiated fast DC apoptosis and significantly modified the transcriptional upregulation of biologically relevant DC cytokines (Sherrid and Hybiske, 2017). Hybiske and Stephens also claimed that both processes, extrusion and lysis, are prevalent among genital as well as LGV biovars of C. *trachomatis* and that they occur at nearly equivalent frequencies (Hybiske and Stephens, 2007), thus objecting the papers of Banks et al. (1970) and Todd and Caldwell (1985).

Recently, studies from Lutter et al. (2013) provided the first insights into the regulation of egress mechanisms in *C. trachomatis*. The group discovered the recruitment of MYPT1, a subunit of myosin phosphatase, to the periphery of the inclusion through interaction with inclusion membrane protein CT228, an apparent central player in the regulation of egress. MYPT1-mediated phosphorylation of myosin light chain II (MLC2) favored extrusion, while the depletion or dephosphorylation of MLC2 favored lysis (Lutter et al., 2013).

The stability of the inclusion membrane depends on the presence of an actin scaffold surrounding it. Hence, in order to exit the host cells through lysis, chlamydiae must be able to disassemble this scaffold. The group of Yang et al. (2015) demonstrated that the cryptic chlamydial plasmid controls lysis. They furthermore demonstrated that plasmid gene protein 4 (Pgp4), a transcriptional regulator of multiple chromosomal genes, is essential for actin depolymerization prior to cell exit and that Pgp4-dependent release is reliant on the chlamydial T3SS (Yang et al., 2015). Furthermore, the previously discussed chlamydial protease CPAF, cleaving intermediate filaments, is believed to also be involved in chlamydial release, however, it is not involved in actin depolymerization (Snavely et al., 2014).

## The Role of Inclusion Membrane Associated Proteins in Fulfilling Chlamydial Metabolic Needs

### Inclusion Membrane Transport Proteins

The inclusion provides shelter to the bacteria by shielding them from the host but on the other hand the inclusion membrane also represents a metabolite barrier (Heinzen and Hackstadt, 1997). It has a neutral pH and is permeable to ions (Grieshaber et al., 2002), yet impermeable to compounds larger than 520 Da (Heinzen and Hackstadt, 1997). In order to effectuate the uptake of metabolites by means of chlamydial nutrient transporters, embedded in the pathogens cell wall, the necessary metabolites need to be present in the inclusion lumen.

Eukaryotic lipids are obtained via vesicular and non-vesicular pathways (as discussed in great detail later in this review). In the former process, also soluble nutrients contained inside the host-derived vesicles could be provided to the bacteria by means of vesicle fusion with the inclusion membrane. However, the composition of the solutes which are present in the lumen of many intracellular vesicles remains unknown and therefore the ability of such metabolites to fulfill the needs of chlamydiae is arguable (Haferkamp, 2017). On the other hand, the inclusion membrane might also contain transport proteins, which mediate the passage of specific solutes. This hypothesis is supported by proteomic analyses of the inclusion membrane, revealing the presence of several host proteins, including membrane proteins of the ER, the Golgi apparatus and the plasma membrane (Saka et al., 2011; Aeberhard et al., 2015; Herweg et al., 2015, 2016). In the following, some examples of inclusion membrane transporters are provided, accompanied with their respective chlamydial body nutrient transporters.

Biotin is a vitamin which functions as a cofactor for multiple carboxylase enzymes. Although some *Chlamydia* species have the enzymatic repertoire for *de novo* synthesis (e.g., *C. pneumoniae*), others do not and thus depend on biotin hijacking (e.g., *C. trachomatis*) or can do both (e.g., *C. psittaci*). Uptake of biotin by the bacteria is mediated by transporter BioY, provided that biotin is present in the inclusion lumen. In case of cells infected with *C. trachomatis*, host SMVT, a biotin transporter, is redirected from the plasma membrane to the inclusion membrane. Interestingly, the host cell expression of SMVT gets upregulated under conditions of increased demand, e.g., due to biotin uptake by the pathogen (Fisher et al., 2012).

Because the genome of *C. trachomatis* lacks most biosynthetic pathways for amino acids (Stephens et al., 1998; Read et al., 2000), the pathogen hijacks most amino acids from the host as well (Bader and Morgan, 1958; Mehlitz et al., 2017). Nevertheless, it appears that *C. trachomatis* synthesizes a small fraction of alanine, aspartate and glutamate *de novo* (Mehlitz et al., 2017). Especially the use of host-derived molecules in providing chlamydiae with tryptophan appears to be crucial. Some species require the host-derived precursor kynurenine for tryptophan synthesis since they only possess part of the biosynthetic pathway (*C. caviae*, *C. felis*, and *C. pecorum*) whilst others require host-derived tryptophan itself because they lack the full biosynthetic pathway (*C. abortus, C.* pneumoniae and *C. psittaci*) (Xie et al., 2002). This dependence of chlamydiae for tryptophan is used by the hosts immune response by employing tryptophan starvation, a IFNγ-driven process, in an attempt to clear chlamydiae. However, tryptophan starvation can either kill the tryptophan auxotrophic *Chlamydia* (Byrne and Beatty, 2012) or alter their gene transcription and metabolism, thereby transforming them into a persistent state (Beatty et al., 1993, 1994). Moreover, genital *C. trachomatis* serovars kept a relict pathway, utilizing indole for tryptophan synthesis, a trait the ocular serovars lost. Interestingly, in this case, the indole is not a product from human host cells but rather from indole-producing microbiota that can co-exist in the genital tract in case of bacterial vaginosis (Ziklo et al., 2016). Hence, strain-specific tropism is also correlated with the ability to hijack indole from a co-existing microbial community (Fehlner-Gardiner et al., 2002). Genome analysis showed that *C. trachomatis* possesses at least one carrier for tryptophan uptake (Bonner et al., 2014). The tryptophan is first shuttled from the host cytosol to the inclusion lumen by means of two components of the human amino acid uptake system: SLC7A5 and SLC3A2 (Aeberhard et al., 2015). These components can form a heterodimer through covalent interactions, which then functions as an amino acid antiporter that prefers large amino acids (Wagner et al., 2001). The latter include tryptophan but also phenylalanine, tyrosine and histidine. Nevertheless, transport of smaller amino acids such as methionine, valine, or leucine is also possible (Kanai et al., 1998; Yanagida et al., 2001).

Glycogen is a multi-branched glucose polysaccharide that represents an important energy storage compound for animals, humans, and bacteria. *C. trachomatis* RBs store glycogen in the inclusion lumen (Gordon and Quan, 1965) and later, during RB-EB transition, tap from this supply to build up an intra-bacterial glycogen stock. In the latter scenario, luminal glycogen gets degraded into glucose-1-phosphate and subsequently converted to glucose-6-phosphate. Chlamydiae rely on the import of phosphorylated sugars because they lack a hexokinase, the enzyme phosphorylating glucose (Gehre et al., 2016). Plausible glucose-6-phosphate transporter have yet been suggested for *C. trachomatis* and *C. pneumoniae* (Schwöppe et al., 2002). A minor amount of the glycogen, present in the lumen of the inclusion, is acquired by translocation in bulk via vesicular pathways. However, the majority of the glycogen in the inclusion lumen is synthesized *de novo* by means of two secreted bacterial enzymes: bacterial glycogen synthase GlgA and branching enzyme GlgB. The required precursor, host-derived UDP-glucose, enters the inclusion via solute carrier SLC35D2, embedded in the inclusion membrane (Gehre et al., 2016). Moreover, *Chlamydia* is shown to induce host hexokinase II, an enzyme known to play a critical role in regulating glucose entry into the cell by catalyzing the first step in glucose metabolism (Al-Zeer et al., 2017).

The group of Wang et al. (2017) demonstrated that *Chlamydia* exploits host-derived transporter proteins glucose transporter proteins 1 and 3 (GLUT1 and GLUT3) to fulfill its carbon source requirements by altering their expression, turnover and localization. Knockdown of these protein, using siRNA, clearly affected chlamydial development. The upregulation of GLUT1 and GLUT3 during chlamydial infection was proven to be dependent on bacterial protein synthesis and *Chlamydia*-induced MAPK kinase activation. Furthermore, GLUT1, but not GLUT3, was proven to be in close proximity to the inclusion membrane throughout the chlamydial life cycle and this proximity was dependent on a brefeldin A-sensitive pathway. Finally, stabilization of GLUT1 through inhibition of host-dependent ubiquitination by chlamydial deubiquitinase effector protein CT868 was explained (Wang et al., 2017).

A Npt1 antiporter is present in the chlamydial cell wall and catalyzes the uptake of host-derived ATP in exchange for bacterial ADP and phosphate (Tjaden et al., 1999; Trentmann et al., 2008). This implies that the presence of ATP in the inclusion lumen is necessary and that ADP and phosphate have to be eliminated. The latter is mandatory since these molecules can outcompete ATP in the binding of Npt1. Delivery of ATP could be completed via vesicle fusion, however, as mentioned previously, whether or not vesicles provide the inclusion with solutes remains unknown. Moreover, the need to remove ADP and phosphate points in the direction of carrier-mediated nucleotide translocation. Therefore, it is assumed that host adenine nucleotide transporters from the ER and/Golgi are inserted in the inclusion membrane, although possible candidates remain unknown. However, remarkably, it appears that inclusion membranes also contain Npt1 (Saka et al., 2011).

### Interactions Between Inclusions and Mitochondria

Mitochondria were found in close association with *C. psittaci* and *C. caviae* inclusions, but this association was not observed in *C. trachomatis* and *C. pneumoniae*-infected cells (Matsumoto et al., 1991; Vanrompay et al., 1996). The translocase of the inner membrane–translocase of the outer membrane (TIMTOM) complex, proven to be involved in the recognition and transport of host mitochondrial proteins into the mitochondria, is essential for *C. caviae* and *C. trachomatis* inclusion biogenesis and the production of infectious progeny. Depletion of this complex disrupts their chlamydial infection (Kokes and Valdivia, 2012; Gurumurthy et al., 2014). The functional significance of these associations might be related to the hijacking of energy metabolites. Although chlamydiae have the capacity to produce ATP, the genes required are only transcribed starting from 6 h post infection. Therefore, energy needed for the early differentiation of EBs to RBs might either come from chlamydial ATP reserves but also from the host (Iliffe-Lee and McClarty, 1999). This hypothesis is supported by the fact that chlamydiae contain mimic-ATP transporters Npt1 and Npt2 in their EB, RB and inclusion membranes, as mentioned before (Tjaden et al., 1999; Saka et al., 2011). However, prevention of apoptosis is also suggested as a reason to why inclusions associate with mitochondria. The release of mitochondrial cytochrome c into the cytoplasm is crucial to induce apoptosis (Yang, 1997). This being said, one of the observed effects of chlamydial effectors is the prevention of mitochondrial cytochrome c release into the host cytoplasm (Fan et al., 1998). Prevention of host cell apoptosis is of great interest for the obligate intracellular pathogen for completion of its life cycle. Other recently discovered chlamydial anti-apoptotic strategies involve the induced expression and increased stabilization of host anti-apoptotic proteins such as Mcl-1 (Rajalingam et al., 2008; Sarkar et al., 2015) and inhibitor of apoptosis proteins IAPs (Prakash et al., 2009), and the upregulation of specific members a group of diverse miRNAs, called apoptomirs. These miRNAs target several pro- as well as anti-apoptotic proteins and thus influence apoptotic signaling pathways. Chowdhury et al. (2017) observed that *C. trachomatis* significantly upregulates miR-30c-5p, which targets tumor suppressor protein p53. Subsequent loss of p53 is of interest for chlamydiae since activation of p53 suppresses the pentose phosphate pathway, which is essential to chlamydial growth (Chowdhury et al., 2017). Moreover, miR-30c-5p also downregulates the major mitochondrial fission regulator, Drp1 and therefore obstructs fission of mitochondria, due to both intrinsic and extrinsic pro-fragmentation stimuli, and the subsequent degradation of the resulting fragments. This preservation of the mitochondrial architecture is beneficial for chlamydiae since the mitochondria represent an essential source of ATP (Chowdhury et al., 2017).

### Interactions Between Inclusions and Lysosomes

Although chlamydiae modify the inclusion membrane to prevent fusion with the endolysosomal pathway, lysosomes reside in close proximity of the inclusion membrane. Therefore, Ouellette et al. (2011) hypothesized the possibility that *Chlamydia* hijack lysosomal amino acids and/or oligopeptides. This hypothesis was proven by the labeling of chlamydiae that were grown inside cells which were administered labeled proteins as an exogenous nutrient source (Ouellette et al., 2011). Moreover, treatment with Bafilomycin A1 (BafA1), an inhibitor of the vacuolar H+/ ATPase that blocks lysosomal acidification, impaired the growth of *C. trachomatis* and C. *pneumoniae*, with more profound effects in the latter. This inhibition of growth was further proven not to be due to changes in lysosomal acidification *per se*, since cathepsin inhibitors also inhibited growth. Finally, Ouellette et al. (2011) showed that EBs contain both amino acid and oligopeptide transporters whilst early after differentiation, RBs predominantly use their oligopeptide transporters in order to acquire oligopeptides from lysosomes. However, later in the infection cycle, *C. trachomatis* uses its amino acid transporters to hijack free cytosolic amino acids, whereas *C. pneumoniae* continues to depend on lysosome-derived oligopeptides (Ouellette et al., 2011).

### Vesicular and Non-vesicular Pathways

**FIGURE 5:**
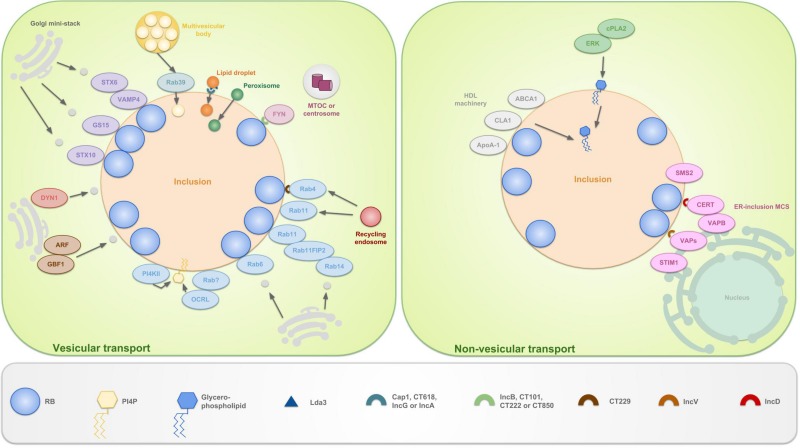
Schematic representation of the vesicular and non-vesicular pathways, employed by *C. trachomatis*. The inclusion selectively interacts with organelles in the peri-Golgi region by means of vesicular and non-vesicular pathways in order to sequester eukaryotic lipids for chlamydial development and survival. These lipids include sphingolipids, cholesterol and glycerophospholipids. The hijacking of lipids derived from the Golgi apparatus is facilitated by the positioning of mini-stacks around the inclusion. Sphingomyelin and cholesterol are attained via interception of exocytic vesicles, fragmented from these mini-stacks. Capturing of vesicles is done by hijacking Golgi-associated Rabs (such as Rab6, 11 and 14), which promote selective interaction and/or fusion between several host vesicles. Rab4 and 11 also mediate interactions with the transferrin slow-recycling pathway in order to acquire iron. Recruitment of these Rabs is done by chlamydial Incs, e.g., CT229 recruits Rab4. Rab11 on his turn recruits Rab11FIP2 and together these recruit Rab14. In addition to trafficking, Rabs also promote vesicle fusion by recruiting lipid kinases such as inositol polyphosphate 5-phosphatase OCRL1, a Golgi-localized enzyme and PI4KIIα. Both produce the Golgi-specific lipid PI4P and enrichment of the latter is considered a strategy to disguise the inclusion as a specialized compartment of the Golgi apparatus. Furthermore, chlamydiae interact with trans-Golgi STX6 and STX10, VAMP4 and GS15. These also regulate the acquisition of nutrients from the Golgi exocytotic pathway. Moreover, GBF1, a regulator of Arf1-dependent vesicular trafficking within the early secretory pathway is employed. Finally, growth of *C. trachomatis* is depending on interactions with DYN1, a large GTPase that induces scission of vesicles from, among others, the Golgi apparatus. Whether or not FYN kinase regulates vesicle-mediated trafficking is currently unknown. However, it is hypothesized that FYN mediates linkage of the inclusion to the microtubule network, thereby intersecting sphingomyelin-containing vesicles that traffic along the microtubules. LDs, peroxisomes and MVBs also represent useful sources for eukaryotic lipids and get translocated fully into the inclusion. LDs are ER-derived storage organelles for neutral lipids or long chain fatty acids. Lda3 gets translocated to the host cytosol after which it links cytoplasmic LDs to the inclusion membrane. Furthermore, it was suggested that IncA might mark the entry sites for LDs at the inclusion membrane. The mechanisms of peroxisome and MVB uptake are still unclear, although Rab39 was shown to participate in the delivery of MVBs to the inclusion. ER derived sphingomyelin on the other hand is acquired via non-vesicular pathways. The existence of ER-inclusion MCSs was shown in which CERT, ER-resident protein VAPB, SMS2 and ER calcium sensor STIM1 are enriched. CERT is proven to be recruited to these MCSs via direct interaction with IncD, which in turn leads to the binding of CERT with VAPB. It is believed that CERT and VAPB participate to the non-vesicular trafficking of ceramide, the precursor of sphingomyelin, from the ER to the inclusion, after which ceramide is further converted into sphingomyelin by SMS2. The role of STIM1 remains unanswered. Besides CERT also IncV is able to interact with VAPs, possibly assisting in ER-inclusion tethering. Another non-vesicular pathway involves the co-opting of the host cell lipid transport system involved in the formation of HDLs. HDL is formed when cholesterol and phospholipids are transported to extracellular ApoA-1 by the lipid binding proteins ABCA1 and ABCG1 and CLA1. ABCA1, CLA1 and ApoA-1 are shown to be localized at the inclusion membrane. Lastly, PI and PC are acquired via another non-vesicular pathway, mediated by ERK and cPLA2.

In addition to employing the nutrient transporters that are embedded in its membrane, the inclusion selectively interacts with organelles in the peri-Golgi niche in order to sequester essential factors for chlamydial development. Despite the fact that chlamydiae are capable in producing the common bacterial lipids, it is imperative for their development and survival that eukaryotic lipids are also present in their membranes. Sphingolipids (Hackstadt et al., 1996), cholesterol (Carabeo et al., 2003), and glycerophospholipids (Wylie et al., 1997) are essential for several processes including but not limited to chlamydial replication, growth, reactivation from persistence and RB to EB re-differentiation. Since chlamydiae lack the required biosynthetic enzymes, sophisticated interaction with various host pathways are exerted to acquire those eukaryotic lipids, involving vesicular and non-vesicular pathways (Figure 5). However, Gilk et al. (2013) observed that cholesterol precursors may be sufficient for enabling *C. trachomatis* infection since *C. trachomatis* growth and inclusion formation were unaffected in cholesterol-free mouse embryonic fibroblasts (Gilk et al., 2013).

#### Vesicular Pathways

##### Golgi apparatus

The Golgi apparatus fragments into mini-stacks during the mid-cycle stages of *C. trachomatis* infection. These fragments surround the inclusion in order to increase the efficiency in delivery of eukaryotic lipids. Furthermore, artificial enhancement of mini-stack formation by depletion of Golgin-84 is proven to increase infectious progeny production (Heuer et al., 2009). However, fragmentation is not imperative to the growth of *C. trachomatis* and to lipid uptake (Gurumurthy et al., 2014). Deubiquitinase activity of the chlamydial effector ChlaDUB1 was recently proven to be linked to the fragmentation of the host Golgi apparatus (Pruneda et al., 2018). Around 12 h post-infection, a “cage” of post-translationally modified microtubules surrounds the inclusion and controls the positioning of Golgi complex mini-stacks around it (Al-Zeer et al., 2014). The post-translational modifications influence microtubule structure and depolymerization rates and particularly include acetylated and detyrosinated tubulin (Peris et al., 2009). Wesolowski et al. (2017) showed that the Inc CT813 controls posttranslational modifications and the positioning of the mini-stacks around the inclusion through the recruitment and activation of host Arf GTPases, Arf1 and Arf4. Interestingly, since Arf GTPases get activated by Arf GEFs through nucleotide exchange but CT813 does not display GEF activity, it is believed that CT813 recruits or cellular GEFs or another unidentified compound that allows CT813 to function as a GEF. The latter hypothesis is the most plausible one since CT813 behaves as a GEF by interacting with Arf-GDP as well as Arf-GTP and, moreover, competes with cellular GEFs *in vitro* (Mueller and Goody, 2016). Moreover, Al-Zeer et al. (2014) suggest that RhoA and ROCK (Rho-associated protein kinase) activity are essential for the recruitment and/or assembly of stable microtubules at the inclusion membrane. Finally, data from the group of Dumoux et al. (2015) mechanistically complemented and extended this model, as they identified *C. trachomatis* effector IPAM as an initiator of microtubule organization around the inclusion through its recruitment of CEP170. As mentioned previously, in addition to microtubule cages, the chlamydial inclusion is also surrounded by a network of actin, which ensures integrity of the inclusion (Kumar and Valdivia, 2008). Interestingly, since CT813 is proven to also be involved in the formation of these actin scaffolds (Kokes et al., 2015), CT813 is suggested to be a master cytoskeletal regulator (Wesolowski et al., 2017).

Sphingomyelin and cholesterol are attained via interception of exocytic vesicles from these fragmented Golgi-mini-stacks, destined for the plasma membrane (Hackstadt et al., 1996; Carabeo et al., 2003) and via multivesicular bodies (see ‘Translocation into the inclusion’) (Beatty, 2006, 2008; Kesley Robertson et al., 2009). In the former acquisitioning strategy, host proteins such as Rab GTPases (specifically, Rab6, Rab11 and Rab14 in case of *C. trachomatis*) (Lipinski et al., 2009; Capmany et al., 2011) and Rab11FIP2 (Leiva et al., 2013), SNARE proteins (Kabeiseman et al., 2013), Arf GTPases (Moorhead et al., 2010; Reiling et al., 2013), the Arf guanine nucleotide exchange factor GBF1 (Elwell et al., 2011), dynamin (Gurumurthy et al., 2014) and FYN kinase (Mital et al., 2010; Mital and Hackstadt, 2011a, b) are involved.

###### Rab GTPases

Several endosome and Golgi-related Rab GTPases, which are master controllers of intracellular trafficking, membrane fusion and organelle identity (Seabra and Wasmeier, 2004; Hutagalung and Novick, 2011), associate with the inclusion membrane. Rab proteins were observed in association with the inclusion membrane in both a species-dependent and species-independent manner, depending on the protein. For example, Rab1, 4, and 11 are recruited to the inclusion membranes of *C. trachomatis*, *C. muridarum* and *C. pneumoniae*. In contrast, Rab6 is recruited to the inclusion membranes of *C. trachomatis* but not those of *C. pneumoniae* or *C. muridarum*, while the opposite is true for Rab10 (Rzomp et al., 2003; Brumell and Scidmore, 2007).

Rab GTPases function in various pathways and the recruitment of different Rabs is suggested to promote selective interaction and/or fusion with several host vesicles containing essential nutrients (Bastidas et al., 2013). Chlamydiae hijack Golgi-associated Rabs (such as Rab6, 11 and 14) to capture exocytic vesicles enriched in endogenously synthesized host lipids (Rzomp et al., 2003). Rab6 and 11 mediate fragmentation of the Golgi into mini-stacks (Heuer et al., 2009; Lipinski et al., 2009) while Rab14 mediates delivery of Golgi-derived sphingomyelin to the inclusion (Capmany and Damiani, 2010; Damiani et al., 2014). On the other hand, Rab GTPases may also be involved in the acquisition of nutrients other than those originated in the Golgi apparatus: Rab4 and 11 mediate interactions with the transferrin slow-recycling pathway in order to acquire iron. Although Rab4 silencing failed to reveal any effect on the pathogen, simultaneous depletion of Rab4 and Rab11 impaired chlamydial growth (Ouellette and Carabeo, 2010).

Chlamydial Incs probably recruit host-derived Rabs through species-specific interactions (Rzomp et al., 2003). For example, the *C. pneumoniae* Inc Cpn0585 interacts with Rab1, 10 and 11 (Cortes et al., 2007). Rab11 on its turn recruits the Rab11 effector, Rab11 family interacting protein 2 (Rab11FIP2) and together they promote the recruitment of Rab14 (Leiva et al., 2013). Since it is unclear for *C. trachomatis* which Inc interacts with Rab11 and since their genome does not encode a protein that is homologous to Cpn0585, binding partners could possibly be detected by performing sequence comparison studies, restricted to the functional regions of Cpn0585 (Cortes et al., 2007). *C. trachomatis* Inc CT229 recruits among others Rab4 (Rzomp et al., 2006). Moreover, the importance of CT229 in forming and maintaining the intracellular niche of *C. trachomatis* is proven by the fact that absence triggers premature inclusion lysis and host cell death (Weber et al., 2017). Moreover, recently, the group of Faris et al. (2019) demonstrated that CT229 recruits multiple Rab GTPases and their cognate effectors to the inclusion and that CT229 redirects and intercepts host clathrin-coated vesicles from the recycling pathway and thereby regulates the trafficking of transferrin and the mannose-6-phosphate receptor.

Incs are a family of T3S effector proteins which are expressed at several time point during the developmental cycle (Nicholson et al., 2003) but primarily early in the infection, when they are important in the escape of the inclusion from the endolysosomal pathway, and at mid-cycle, when they are essential in the acquisition of host-derived nutrients (Moore and Ouellette, 2014). Incs are identified by one or more N-terminal bilobed hydrophobic domains, composed of two closely spaced membrane-spanning regions that are separated by a short hairpin loop (Bannantine et al., 2000). Furthermore, their amino terminus and/or carboxyl terminus is predicted to extend into the cytoplasm of the host cell (Rockey et al., 2002; Hackstadt, 2012). Finally, the N-terminal type 3 secretion signal that allows the secretion and subsequent insertion of the proteins into the inclusion membrane is also an identifier (Bauler and Hackstadt, 2014; Moore and Ouellette, 2014). During a bioinformatics study by Lutter et al. (2012) the following numbers of Incs were identified: 55 in *C. trachomatis*, 68 in *C. felis*, 92 in *C. pneumoniae*, 79 in *C. caviae* and 54 in *C. muridarum*. Furthermore, Inc homologues were compared across these five species and a core set of 23 Incs was identified as shared among all, possibly representing proteins involved in conserved interactions between chlamydiae and the host. On the other hand, the diversification of Incs between these species suggests the involvement of certain Incs in unique pathogenic pathways. Moreover, genomic expansion of Incs was identified in *C. pneumoniae*, *C. caviae* and *C. felis* but not *C. trachomatis* or *C. muridarum*. Finally, Lutter et al. (2012) pointed out that, besides the previously mentioned Pmps and Tarp, also some *C. trachomatis* Incs clustered the different biovars, thus suggesting that these proteins may also contribute to tissue tropism. Furthermore, also the group of Almeida et al. (2012) pointed out that subtle variations in the amino acids of a subset of *C. trachomatis* Incs and in their expression might contribute to the invasive character of *C. trachomatis* LGV strains. The 50 genes coding for Incs represent approximately 6% of the coding capacity of *C. trachomatis* (Stephens et al., 1998). Given the highly reduced genome of chlamydiae, Incs thus serve an important function (Moore and Ouellette, 2014).

In addition to trafficking, Rabs also promote vesicle fusion by recruiting lipid kinases such as the inositol polyphosphate 5- phosphatase OCRL1 (also known as Lowe oculocerebrorenal syndrome protein), a Golgi-localized enzyme, and phosphatidylinositol-4-kinase type IIα (PI4KIIα). Both produce the Golgi-specific lipid phosphatidylinositol-4-phosphate (PI4P). The enrichment of PI4P is considered a strategy to disguise the inclusion as a specialized compartment of the Golgi apparatus (Moorhead et al., 2010).

###### SNARE proteins

Vesicular pathways might also be regulated by the recruitment of host soluble N-ethyl-maleimide-sensitive factor attachment receptor (SNARE) proteins, which are key components of the intracellular fusion machinery (Südhof and Rothman, 2009). Chlamydiae interact, among others, with trans-Golgi SNARE proteins syntaxin 6 (STX6) (Moore et al., 2011) and STX10 (Lucas et al., 2015), vesicle-associated membrane protein 4 (VAMP4) (Kabeiseman et al., 2013) and GS15 (also known as BET1L) (Pokrovskaya et al., 2012). These regulate the acquisition of nutrients from the Golgi exocytic pathway. The group of Kabeiseman et al. (2013) observed that knockdown of VAMP4 prevented localization of STX6 to the chlamydial inclusion, 1 year later, proved that STX6 is trafficked to the chlamydial inclusion by means of its YGRL signal sequence, after which it interacts with VAMP4 and remains on the inclusion membrane (Kabeiseman et al., 2014).

Remarkably, chlamydiae also use SNARE motifs to inhibit vesicle fusion through molecular mimicry by Incs. At least three Incs contain SNARE-like motifs, enabling them to act like SNARE proteins and limit fusion with compartments that contain VAMP3, VAMP7, or VAMP8, all three SNAREs involved in endosomal trafficking (Delevoye et al., 2008; Kokes and Valdivia, 2012; Ronzone and Paumet, 2013). These Ins include IncA (also known as CT119), InaC (also known as CT813) and an Inc, acting on microtubules (IPAM or CT223) (Delevoye et al., 2008). IncA is exposed on the cytosolic face of the *C. trachomatis* inclusion membrane (Hackstadt et al., 1999) and displays two coiled-coil domains, which show high homology with SNARE proteins. IncA inhibits host endocytic SNARE-mediated membrane fusion for target SNAREs, positioned on the same membrane as IncA (Paumet et al., 2009; Ronzone and Paumet, 2013). However, IncA is also involved in the induction of inclusion homotypic fusion (Hackstadt et al., 1999; Suchland et al., 2000). In host cells which are multiply infected with *C. trachomatis*, the inclusions fuse to form a single large vacuole (Blyth and Taverne, 1972; Ridderhof and Barnes, 1989). Since the absence of IncA correlates with the formation of multilobed non-fusogenic inclusion bodies, homotypic vesicle fusion of inclusions relies on IncA (Suchland et al., 2000; Pannekoek et al., 2005). Moreover, IncA-negative strains were studied by the group of Geisler et al. (2001) who showed that non-fusogenic clinical isolates induced less severe clinical signs of infection with low *Chlamydia* recovery. The mechanism by which IncA regulates the delicate balance between blocking lysosomal membrane fusion and promoting homotypic inclusion fusion remains elusive. However, Ronzone and Paumet proved that although both coiled-coil domains of IncA are each on their own capable to inhibit SNARE-mediated fusion, cooperation between these two coiled-coil domains is essential in mediating IncA multimerization and homotypic membrane fusion.

###### Arf GTPases and GBF1

The hijacking of host sphingomyelin from Golgi is a Brefeldin A (BFA)-sensitive vesicular trafficking pathway (Hackstadt et al., 1996; Elwell et al., 2011). Elwell et al. (2011) showed that *C. trachomatis* selectively opts only one of the three known BFA targets: GBF1, a regulator of Arf1-dependent vesicular trafficking within the early secretory pathway. The Arf1/GBF1-dependent pathway of sphingomyelin acquisition is proven to be crucial for the growth of the inclusion membrane, yet not necessary for bacterial amplification (Elwell et al., 2011).

###### Dynamin

Dynamin is a large GTPase that induces scission of vesicles from, among others, the Golgi apparatus and that is necessary for the formation of both clathrin-coated and non-clathrin-coated vesicles from the *trans*-Golgi network. It appears that dynamin is required for the growth of *C. trachomatis* because it is essential for homotypic fusion of the *C. trachomatis* inclusions. Moreover, suppressing of dynamin activity leads to the disruption of lipid trafficking into *C. trachomatis* inclusions and dynamin-mediated lipid acquisition is proven to be unrelated to Golgi-fragmentation. Finally, dynamin activity is shown to be necessary for normal re-differentiation from RBs to EBs (Gurumurthy et al., 2014).

###### FYN kinase

Mital and Hackstadt identified host protein FYN kinase, part of the SFKs, as a regulator of sphingomyelin acquisition in *C. trachomatis* since depletion of FYN kinase decreased sphingolipid retention by both the inclusion and EBs. However, since infectious progeny was still produced, despite this depletion, the FYN kinase pathway was assumed to be redundant to other lipid trafficking pathways (Mital and Hackstadt, 2011b). Moreover, as mentioned earlier, Mital et al. (2010) have shown that for *C. trachomatis*, four Incs (IncB, Inc101, Inc222, and Inc850) co-localized with the active FYN and other active Src kinases in discrete cholesterol-rich microdomains at the point of centrosome–inclusion contact, making it likely that these Incs participate in transport of the inclusion along microtubules. Whether FYN kinase regulates vesicle-mediated trafficking from the Golgi apparatus and/or MBVs to the chlamydial inclusions and whether Fyn plays a role in cholesterol acquisition is currently unknown. However, it is hypothesized that FYN mediates linkage of the inclusion to the microtubule network, thereby intersecting sphingomyelin-containing vesicles that traffic along the microtubules (Mital and Hackstadt, 2011a).

###### CteG

Very recently, the group of Pais et al. (2019) identified CT105 as a T3S effector of *C*. *trachomatis*. CT105 was shown to localize to the Golgi-apparatus and the plasma membrane of infected host cells. Moreover, they showed that CT105 can modulate eukaryotic vesicular trafficking. Because CT105 is the first *Chlamydia* effector proven to associate with the Golgi complex, they named the protein CteG (for *C*. *trachomatis* effector associated with the Golgi). However, no information is gathered yet on the function and subcellular targeting mechanisms of CteG as well as its diversity and specificity within *C*. *trachomatis* and among *Chlamydia* species (Pais et al., 2019).

##### Translocations into the inclusion

###### Lipid droplets

Lipid droplets (LD) are ER-derived storage organelles for neutral lipids or long chain fatty acids (Martin and Parton, 2006; Cocchiaro et al., 2008). Three *C. trachomatis* LD-associated proteins were identified: Lda1, Lda2, and Lda3 (Kumar et al., 2006). The roles of Lda1 and Lda2 are unknown to date. Contrarily, ectopical expression of Lda3 shows that it has tropism for both LDs and the inclusion membrane, indicating its potential to act as a molecular bridge between them (Cocchiaro et al., 2008). Cocchiaro et al. (2008) proposed a model in which secreted Lda3 binds to LDs in the vicinity of the inclusion, where after the Lda3-tagged LDs then dock with the inclusion membrane by binding to a hypothetical chlamydial protein (IncX). Next, the inclusion membrane would invaginate to deliver an intact LD into the inclusion lumen, where it intimately associates with RBs. Furthermore, since IncA cofractionated with LDs, accumulated in the inclusion lumen and partially colocalized with intraluminal LDs, it was suggested that IncA might mark entry sites for LDs at the inclusion membrane. Moreover, Lda3 might also participate in the hijacking of host LDs by promoting the removal of the LD protective coat protein, adipocyte differentiation related protein (ADRP) (Cocchiaro et al., 2008). Moreover, Saka et al. (2015) noted that IncG (CTL00373/CT118), Cap1 (CTL0791/CT529), CTL0882 (CT618) also associated with LDs when ectopically expressed in host cells. They thus speculated that the expression of these Incs may again represent a bacterial strategy to promote the previously reported close association of these organelles with inclusion membranes (Saka et al., 2015). Furthermore, LD-associated proteins might also influence chlamydiae. The human acyl-CoA carrier, acyl-CoA-binding domain-containing protein 6 (ACBD6), for example affects the bacterial acyltransferase activity of CT775, thus the formation of phophatidylcholine in *C. trachomatis* (Soupene et al., 2015).

Excess cholesterol is esterified by acyl CoA transferase (ACAT) prior to packaging in LDs. Expression of ACAT1 is increased in HP-1 cells infected with *C. pneumoniae*, resulting in a higher level of esterified cholesterol (Liu et al., 2010). Moreover, a decreased in cholesterol efflux, discussed later in section ‘HDL biogenesis,’ results in cholesterol accumulation within the host cell (Samanta et al., 2017).

The observation that the LDs do not accumulate in the inclusion lumen led to the suggestion that they are consumed, either for energy generation and/or as a source of fatty acids for lipid biosynthesis (Cocchiaro et al., 2008). However, the group of Sharma et al. (2018) states that it is not the LDs *per se*, but the availability of fatty acids in the host cells that contribute to the growth and development of *C. trachomatis*. It is believed that *C. trachomatis* CT149 might liberate cholesterol from LDs for use of bacteria since LDs store cholesterol esters. CT149 was localized inside the inclusion lumen by means of antibodies (Peters et al., 2012; Samanta et al., 2017). It is a putative carboxylic esterase containing a cholesterol recognition consensus sequence and two GXSXG cholesterol esterase motifs. Cholesterol esterase activity of recombinant CT149 was proven *in vitro*. Moreover, cholesterol ester levels decreased and free cholesterol levels increased when ectopically expressing CT149 in HeLa cells (Peters et al., 2012; Samanta et al., 2017).

###### Peroxisomes

Three-dimensional fluorescence microscopy revealed that peroxisomes are translocated into the chlamydial inclusion as well, where they are located adjacent to the bacteria. The mechanism of peroxisome uptake, however, is still unclear. Boncompain et al. (2014) have shown that peroxisomes are not essential for bacterial development *in vivo* since chlamydiae are able to multiply and form infectious progeny in host cells, deficient for peroxisome biogenesis.

###### Multivesicular bodies

Multivesicular bodies (MVBs) are heterogeneous late endocytic organelles essential for the sorting and processing of proteins and lipids that are destined for lysosomal degradation, recycling to the Golgi, or plasma membrane exocytosis (Denzer et al., 2000; Piper and Luzio, 2001; Woodman and Futter, 2008). Chlamydiae use the MVBs as an additional lipid (sphingolipids, phospholipids, and cholesterol) source by translocating the MVBs into the inclusion (Beatty, 2006; Gambarte Tudela et al., 2015). Different MVB markers, such as CD63 and lysobisphosphatidic acid (LBPA), localize to the *C. trachomatis* inclusion lumen (Beatty, 2006). MVBs migrate along microtubules toward the inclusion and Rab39, which labels a subset of late endosomal vesicles, mainly MVBs, participates in the delivery of the MVBs to the inclusion (Gambarte Tudela et al., 2015). However, the chlamydial effectors involved in the transport of MVB into the inclusion lumen are unknown (Gambarte Tudela et al., 2015; Dumoux and Hayward, 2016).

#### Non-vesicular Pathway

One chlamydial non-vesicular mechanism is the use of lipid transporters such as, among others, host ceramide endoplasmic reticulum transport protein (CERT) (Derré et al., 2011; Elwell et al., 2011). Sphingomyelin synthase 2 (SMS2), which is recruited to the inclusion, probably converts the transported ceramide into sphingomyelin (Elwell et al., 2011). Other chlamydial non-vesicular mechanisms include the use of members of the high-density lipoprotein (HDL) biogenesis machinery and the activation of phospholipase A2 and ERK in order to deliver respectively host phosphatidylcholine (Cox et al., 2012) and glycerophospholipids (Su et al., 2004). All aforementioned non-vesicular mechanisms will be discussed in more detail in the following sections.

##### CERT/VAPB/IncD and SMS2

Host-myelin is essential for progeny production and inclusion biogenesis (Van Ooij et al., 2000; Kesley Robertson et al., 2009). Earlier, the use of vesicular pathways to transport sphingomyelin-containing vesicles from the Golgi apparatus to the inclusion was explained. However, BFA-mediated inhibition of vesicular transport shows no effect on the production of infectious progeny (Hackstadt et al., 1996; Hatch and Mcclarty, 1998). This observation suggests the existence of non-vesicular pathways, fulfilling the pathogens need for host-myelin.

*Chlamydia trachomatis* inclusions are shown to be covered with multiple patches of endoplasmic reticulum (ER) at a distance of 10–20 nm from the inclusion membrane (Giles and Wyrick, 2008; Derré et al., 2011; Dumoux et al., 2012). Since zones of close apposition (10-50 nm) between two organelles are usually defined as Membrane Contact Sites (MCSs) (Levine and Loewen, 2006), the points of contact between the ER and the *C. trachomatis* inclusion membrane were named ER-Inclusion MCSs (Derré et al., 2011). Several proteins, specifically enriched at ER-inclusion MCSs of *C. trachomatis* have been identified.

The ceramide transfer protein CERT is a functional component of ER-Golgi MCSs, involved in the non-vesicular transfer of ceramide from the ER to the Golgi (Hanada et al., 2009). CERT is proven to be recruited to the ER-inclusion MCS for *C. trachomatis* infections via direct binding of IncD to the CERT Pleckstrin homology (PH) domain (Derré et al., 2011; Agaisse and Derré, 2014). Amino acid substitution in IncD of ocular and LGV strains of *C. trachomatis* are believed to be involved in the enabling of tissue tropism (Borges et al., 2012; Sugiki et al., 2012). Furthermore, Derré et al. (2011) showed that the binding of IncD to CERT mediates the CERT FFAT motif-dependent recruitment of the ER-resident protein VAPB. However, it remains unclear whether the IncD-CERT-VAPB interaction is sufficient to translocate the ER to the inclusion surface (Derré et al., 2011). Besides CERT, the ER calcium sensor STIM1 is highly enriched at the ER-inclusion MCSs and both proteins colocalize at all stages of the developmental cycle (Agaisse and Derre, 2015). However, while CERT or VAPB depletion affects *C. trachomatis* growth (Derré et al., 2011; Elwell et al., 2011), STIM1 depletion does not, leaving the functional role of the latter unexplained (Agaisse and Derre, 2015). Moreover, Stanhope et al. (2017) recently showed that also IncV is able to interact with VAPs, possibly assisting in ER-inclusion tethering.

In the light of the proposed non-vesicular pathway to supply the inclusion with host-myelin, the recruitment of CERT at the ER-Inclusion MCSs represents a plausible strategy to traffic ceramide from the ER to the inclusion. However, in this scenario the hijacked ceramide still needs to be converted into sphingomyelin. Indeed, sphingomyelin synthase 2 (SMS2) also gets recruited to the inclusion membrane (Derré et al., 2011; Elwell et al., 2011). Moreover, Elwell et al. (2011) hypothesized that sphingomyelin, acquired by non-vesicular transport, is essential for *C. trachomatis* amplification whilst sphingomyelin acquired by vesicular transport is crucial for inclusion membrane expansion and stability (Elwell et al., 2011).

The involvement of CERT in sphingomyelin uptake has also been studied for the zoonotic pathogen *C. psittaci*, although much less extensively compared to *C. trachomatis*. *C. psittaci* also recruits CERT to its inclusion, however, it can also exploit sphingomyelin pathways independent of CERT. Nevertheless, chemical inhibition and CRISPR/Cas9-mediated knockout of CERT affected several stages of the infection including inclusion growth and infectious progeny formation, thus proving that CERT is imperative to *C. psittaci* (Koch-Edelmann et al., 2017).

##### HDL biogenesis

The high-density lipoprotein (HDL) biogenesis machinery is involved in cholesterol and phospholipid efflux. In this process, the lipid binding proteins ATP-binding cassette transporters A1 and G1 (ABCA1, ABCG1), and CLA1 transport the cholesterol and phospholipids toward extracellular ApoA-1 in order to form HDL (Samanta et al., 2017). Interestingly, during chlamydial infection, ABCA1, CLA1, and ApoA-1 localize to the inclusion membrane and both CLA1 and ApoA-1 are found in discrete foci within the inclusion lumen (Cox et al., 2012). Furthermore, the in the inclusion accumulated ApoA-1 co-localized with pools of phosphatidylcholine. Cox et al. (2012) demonstrated that siRNA knockdown of ABCA1 in HeLa cells prevented the growth of *C. trachomatis* and that pharmaceutical inhibitors of ABCA1 and CLA1 transporter activity also inhibited the recruitment of phospholipids to the inclusion, preventing chlamydial growth. These results thus suggested that *C. trachomatis* exploits the host cell lipid transport system involved in the formation of HDL to acquire lipids that are necessary for growth, although the mechanism is not clear (Cox et al., 2012). On the other hand, *C. pneumoniae* downregulates ABCA1 on a translational and a post-translational level (by means of microRNA miR-33), thus targeting cholesterol and phospholipid efflux as a mechanism to further increase intracellular levels (Korhonen et al., 2013; Sun et al., 2014; Zhao et al., 2014).

##### Phospholipase A2 and ERK

Phosphatidylinositol (PI) and phosphatidylcholine (PC), two eukaryotic glycerophospholipids which are present in purified EBs, are also acquired from the host cell through a non-vesicular transport pathway, mediated by ERK and the cytosolic phospholipase A2 (cPLA2). It is believed that chlamydiae actively manipulate the host ERK-cPLA2 signaling pathway since the activation of ERK as well as cPLA2 is reliant on chlamydial amplification and limited to chlamydia-infected cells (Su et al., 2004). Chlamydiae modify the sequestered glycerophospholipids by replacing the non-branched chain fatty acids by *Chlamydia*-derived branched chain fatty-acids, which is in contrast to cholesterol and sphingomyelin that do not get modified (Wylie et al., 1997).

## Molecular Genetic Tools to Study Infection Biology

The function of many chlamydial proteins, crucial for chlamydiae on their way from outside to inside the host cell, remains to be discovered. Studying the role of chlamydial proteins in pathogenesis and virulence has long been challenging because of difficulties related to the manipulation of chlamydial genes (Keb et al., 2018). The transformation of the obligate intracellular chlamydiae with exogenous DNA is a troublesome process because host cell and bacterial membranes represent barriers to reagents. Therefore, *Chlamydia* genetics will likely never reach the tractability level of free-living bacteria (Rahnama and Fields, 2018). In addition, extracellular EBs are not transformation competent, adding a further level of complication (Beare et al., 2011; Rahnama and Fields, 2018). Moreover, because of the considerable reduction of chlamydial genomes in time, interference of functions by using homologues genes from other organisms is often impossible (Brothwell et al., 2016). Nevertheless, recent developments in molecular tools for the genetic manipulation of chlamydiae are overcoming the hurdles that used to impede research on the chlamydial developmental cycle. In what follows, the major advances in genetic engineering techniques in order to study *Chlamydia* infection biology are discussed.

### Transformation Through Electroporation

Besides their highly reduced yet conserved genome of ca. 1 Mb, most *C. trachomatis* isolates carry a cryptic plasmid of 7.5 kb, encoding eight genes (Stephens et al., 1998; Seth-Smith et al., 2009). However, despite the ability of using this plasmid as a vector, genetic manipulation of chlamydiae has long been challenging (Clarke, 2010). The first successful artificial transformation of chlamydiae with recombinant plasmids took place in the year 1994 when electroporation was proven to be able to introduce DNA into the EBs of *C. trachomatis*. The source of DNA for these experiments was a plasmid called pPBW100, which was a chimera between the cryptic plasmid of *C. trachomatis* and the *Escherichia coli* plasmid pBGS9. To select directly for *C. trachomatis* carrying pPBW100, an in-frame gene fusion between the chlamydial promoter P7248 and a promoterless chloramphenicol-resistance cassette was incorporated into the plasmid. After treatment with chloramphenicol of cultured cells, infected with the electroporated EBs, pPBW100 was detected inside the inclusions. Moreover, expression of the resistance cassette was developmentally regulated and occurred during the early stages of RB development. However, the expression of the cassette was mainly transient (Tam et al., 1994). Years later, Binet and Maurelli (2009) tried to transform *C. psittaci* EBs in an attempt to construct variants by homologous recombination. The single rRNA operon was targeted with a synthetic 16S rRNA allele, harboring nucleotide substitutions that, among other, impart resistance to kasugamycin (Ksm) and spectinomycin (Spc). They succeeded in their aim since double resistance and replacement of the 16S rRNA gene were observed (Binet and Maurelli, 2009). The proof-of-concept that was achieved by all of the before mentioned research demonstrated the potential to artificially transform chlamydiae and turned out to be the cornerstone for later developed techniques.

### The Discovery Lateral Gene Transfer and Its Use in Molecular Genetic Tools

The discovery of lateral gene transfer (LGT) via natural transformation, occurring in chlamydiae opened up new perspectives for the development of molecular genetic tools (DeMars et al., 2007; Gomes et al., 2007; DeMars and Weinfurter, 2008; Jeffrey et al., 2010). Intracellular intra- and interspecies LGT was shown to be possible, provided that the involved bacteria cohabitated in a single inclusion (Jeffrey et al., 2013). New techniques were introduced, using LGT for passing mutations from one strain to another, thus enabling genotype–phenotype association studies. Nguyen and Valdivia for example chemically mutated *Chlamydia* and then mapped the underlying genetic lesions by using whole-genome sequencing (WGS) as well as LGT within infected cells. More specifically, the alkylating agent ethyl methyl sulfonate (EMS) was used to mutate Rifampin resistant (Rif^R^) *C. trachomatis* RBs after which the resulting progeny was grown in cell cultures until visible plaques were formed. Common genetic lesions of mutants, sharing the same plaque morphotype, were detected through WGS. Next, genotype-phenotype linkage was studied by coinfecting cell cultures with the Rif^R^ mutants and wild-type Spc^R^ strains and subsequently selecting recombinant LGT-derived Rif^R^ Spc^R^ strains in the presence of rifampin and spectinomycin. Finally, recombinant progeny displaying the desired morphotype was analyzed using targeted DNA sequencing in order to detect segregated individual mutations that were present in the parental mutant strain (Nguyen and Valdivia, 2012). An analogous technique was used by Brothwell et al. (2016) who screened a library of 4,184 EMS-mutagenized *C. trachomatis* isolates for temperature-sensitive (TS) mutants. These mutants only displayed normal development at physiological temperature (37°C). Direct genotype-phenotype linkage was impossible because all but one of the TS mutants contained multiple mutations. Therefore, TS mutants were coinfected and recombinant progeny, growing at the non-permissive temperature of 40°C, was selected for. Subsequent targeted sequencing revealed that the progeny contained all of the mutations present in one TS parent, with the exception of one allele (GltX^Q487*^) (Brothwell et al., 2016).

### Stable Transformation Using a Chimeric Plasmid, Based on the Chlamydial Cryptic Plasmid

Wang et al. (2011) developed the first plasmid-based stable transformation technique for C. trachomatis, based on penicillin selection. Importantly, the technique relied on a calcium chloride (CaCl_2_) treatment of the EBs, rendering them competent (Wang et al., 2011). The group designed a penicillin-resistance gene-containing shuttle vector, based on the chlamydial plasmid and the *E. coli* plasmid pBR325 and able to replicate in both species. Furthermore, they demonstrated the effectiveness and reproducibility of the technique by engineering a penicillin-resistant strain of *C. trachomatis*, expressing GFP (Wang et al., 2011). Results from Wang et al. (2011) presented a turning point in the field of chlamydial genetics, inspiring researchers to start analyzing the importance of the chlamydial plasmid genes in infection biology as well as to use this plasmid in the design of innovative expression vector platforms (Agaisse and Derré, 2013; Ding et al., 2013; Gong et al., 2013; Johnson and Fisher, 2013; Song et al., 2013; Wickstrum et al., 2013). Finally, work by the team of (Gérard et al., 2013) and more recently (Shima et al., 2018) proved the efficacy of plasmid-based genetic tools on *C. pneumoniae* (Gérard et al., 2013).

### *Chlamydia*-Modified TargeTron^TM^

The evidence of transformable *C. trachomatis* opened up doors to try modify pre-existing genetic engineering techniques for use in *Chlamydia*. Johnson and Fisher therefore modified the TargeTron^TM^ technology for use in *C. trachomatis*. This technology is based on the use of group II introns for targeted insertional disruption of genes. None of these introns have been described for *Chlamydia* but these do occur in ca. 25% of bacterial genomes. There are known to move between genes via a retrotransposition mechanism, regulated by an intron encoded protein (IEP) that possesses RNA maturase, endonuclease, and reverse transcriptase activity (Lambowitz and Zimmerly, 2004; Johnson and Fisher, 2013). According to Johnson and Fisher, the intron (designated as EBS2, EBS1, and δ) recognizes the sequence of the target gene (designated as IBS2, IBS1, and δ’), after which it inserts between the IBS1 and δ’ sites of the target gene. Although insertion of the intron in DNA is a stable process, RNA transcripts remain wild-type and no gene functions are lost due to the splicing out of the intron from the RNA after transcription (Johnson and Fisher, 2013). Perutka et al. (2004) proved that by engineering the EBS1, EBS2, and δ intron sequences, the intron Ll.LtrB from *Lactococcus lactis* can be targeted to new genes of interest. Moreover, they showed that, by removing the Ll.LtrB IEP gene from the intron and expressing it in *trans*, restorage of gene function via post-transcriptional RNA splicing can be avoided. Interestingly, by removing the IEP from the intron, room is created for other genes such as selection cassettes. Perutka et al. (2004) supplied *Chlamydia* with a plasmid that contained both the intron and the IEP gene and subsequent expression of the IEP enabled the insertion of the intron into a target gene. Next, removal of the donor plasmid after intron insertion led to the depletion of IEP and thus the prevention of intron splicing. By consequence, the established intron insertions resulted in gene inactivation (Perutka et al., 2004; Johnson and Fisher, 2013). The latter principle later was marketed by Sigma, who called it TargeTron^TM^ technology. The TargeTron^TM^ vector platform was subsequently modified by Johnson and Fisher, who placed a chlamydial promotor upstream of the intron and inserted an ampicillin-resistance gene into the intron to allow for ampicillin selection. As proof of principle in *C. trachomatis*, Johnson and Fisher used the modified TargeTron^TM^ technology to inactivate the chromosomal gene, encoding IncA, the previously discussed protein that regulates homotypic vesicle fusion. Cells, multiply infected with the resulting IncA(-) mutants, demonstrated the presence of non-fusogenic inclusions, as is also observed for the naturally occurring mutants. Further proof of IncA knock-down was provided by means of Western blotting (Johnson and Fisher, 2013). Moreover, studies, using the insertion mutants in well-established mouse infection models, proof the stability of the intron insertion in an *in vivo* setting (Lowden et al., 2015). In addition, successful insertion of an additional marker *aadA*, a spectinomycin-resistance gene, in *Chlamydia* resulted in the production of targeted double mutants and the ability to use gene complementation (Lowden et al., 2015). However, despite the promising results, specific insertional mutagenesis of genes at the 3′ end of operons is difficult because of the polar effects on the operon. Moreover, the system is based on proprietary algorithms, limiting integration to sites which are evaluated to be efficient.

### Fluorescence-Reported Allelic Exchange Mutagenesis (FRAEM)

Homologous recombination depends on a recombinant vector, carrying the desired modifications. Introduction of this plasmid in the cell results in the exchange of nucleotides with the genome. Finally, elimination of the vector, now containing the original and intact gene, is essential to obtain a change in the phenotype of the cell (Binet and Maurelli, 2009). Binet and Maurelli were the first to achieve the removal of a recombinant vector in *Chlamydia*. They constructed a conditionally replicative plasmid by placing *pgp6*, which is located on the native cryptic chlamydial plasmid and controls plasmid maintenance and inheritance (Gong et al., 2013; Song et al., 2013), under the control of an anhydrous tetracycline (aTet)-inducible genetic circuit. Subsequently they proved the efficacy of this conditionally replicative plasmid by targeting and successfully exchanging *C. trachomatis trpA* for a 2.2 Kb cassette, encoding both β-lactamase and green fluorescent protein (GFP). This was achieved by flanking the cassette with 3 Kb of DNA that are homologous to the sequences upstream and downstream of *trpA*. After allelic exchange, the absence of aTet led to the elimination of the vector. Moreover, the introduction and subsequent elimination of the vector could be observed in real-time thanks to the presence of a mCherry gene on the vector backbone. Successful introduction led to the presence of dual-fluorescent transformants after which elimination of the vector in the absence of aTet resulted in cells that were only positive for GFP. Target versatility of FRAEM was studied by repeating this technique for chlamydial genes *ctl0063*, *ctl0064*, and *ctl0065*. Subsequent WGS on the resulting mutants proved the efficacy and specificity of FREAM on *Chlamydia* (Mueller et al., 2016).

### Floxed-Cassette Allelic Exchange Mutagenesis

FRAEM enables chromosomal gene deletion by inserting a selection cassette, which encodes antibiotic resistance and GFP. However, as already mentioned, the insertion of cassettes in polycistronic operons, which are common in the chlamydial genome, could lead to polar effects. Indeed, FRAEM-mediated deletion of *Chlamydia trachomatis tmeA*, a gene that is transcribed as an operon with *tmeB*, negatively impacted the expression of the latter (Mueller and Fields, 2015; McKuen et al., 2017; Mueller et al., 2016). Therefore, Keb et al. (2018) adapted FRAEM technology in order to create markerless gene deletions by using a *gfp-bla* cassette, flanked by *loxP* sites; called the floxed insertion cassette, in combination with transient production of Cre by expression of the latter from a suicide plasmid pSUmC template. Using the adapted FRAEM technology they succeeded in generating a floxed-cassette *tmeA C. trachomatis* mutant. Next, production of Cre led to the excision of the cassette, after which Cre was depleted through curing of the suicide plasmid encoding it. The latter was accomplished by cultivation under non-selective conditions. As a result, the accomplished *tmeA* mutant did express TmeB. Besides being useful in deletion studies within polycistronic operons, these markerless gene deletions limit artificial effects of a selectable marker on chlamydial fitness and have the ability to excise large regions of chromosomal DNA. The latter is very useful for characterizing the function and contribution to virulence and tissue tropism of chromosomal regions such as the highly polymorphic plasticity zones which contain the previously discussed tryptophan biosynthesis genes, or the family of expanded Pmp, also mentioned in this review (Fehlner-Gardiner et al., 2002; Gomes et al., 2006; Keb et al., 2018; Valdivia and Bastidas, 2018). Furthermore, this technique enables the deletion of non-coding RNAs and small RNAs. Research on these gene regulatory elements in *Chlamydia* is limited to date (Valdivia and Bastidas, 2018).

### CRISPRi

According to Ouellette: “one key tool missing from the chlamydial genetic toolbox is the ability to create conditional knock-outs of a target gene via inducible repression or other means” (Ouellette, 2018). Since it is assumed that most of the chlamydial genes are essential to the bacteria’s infection biology because of the greatly reduced chlamydial genome, conditional knock-outs are crucial in studying the function of these genes. Therefore, recently, Ouellette described an innovative CRISPR interference (CRISPRi) technique that uses the catalytically inactive Cas9 variant (dCas9) of *Staphylococcus aureus* to inducibly and reversibly repress gene expression in *C. trachomatis* (Ouellette, 2018). CRISPRi is predicated on the ability of dCas9, to recognize its cognate guide RNA and bind a target sequence without cleaving it. If binding occurs near the 5′ end or in the promoter region of a gene of interest, transcription will be sterically hindered. Furthermore, by transforming cells with a vector on which the expression of the guide RNA is constitutive whilst the expression of dCas9 is controlled by an inducible promoter, inducible knock-out of a specific target gene can be achieved (Qi et al., 2013; Ouellette, 2018). Ouellette successfully demonstrated the use of this single plasmid system for CRISPRi in *Chlamydia*, targeting the expression of IncA (Ouellette, 2018).

## Concluding Remarks

Although substantial knowledge is gathered on the molecular host–pathogen interactions *Chlamydiae* employ in order to survive and grow, it is clear that far from all pathways have been fully characterized to date. For example, far from all binding partners, involved in the attachment of the pathogen to the host cell, have been identified yet. Furthermore, research mainly focused on actin-dependent internalization of the EB, thus data on actin-independent internalization is scarce. Moreover, although numerous pathways to hijack host cell metabolites have been described, a more detailed study on these processes is necessary in order to fill in remaining scientific gaps and discover new essential and/or redundant pathways. Also, the understanding on the mechanism of chlamydial release from infected host cells is limited. Since the survival and proliferation of chlamydiae depends on all of these selective interactions, more extensive knowledge hereof would represent a powerful tool in the design or optimization of antimicrobials and vaccines. Fortunately, recent advances in the development of molecular genetic tools are enabling the scientific community to start analyzing chlamydial infection biology in more depth. By studying the chlamydial pathogenesis and life cycle, prophylactic and therapeutic strategies to battle this pathogen will gain efficiency.

## Author Contributions

AG analyzed all relevant literature and wrote the manuscript in consultation with NS and DV.

## Conflict of Interest

The authors declare that the research was conducted in the absence of any commercial or financial relationships that could be construed as a potential conflict of interest.
